# Supplementary material for: Association between maternal vitamin D deficiency and small for gestational age: evidence from a meta-analysis of prospective cohort studies
Source: BMJ Open. 2017 Aug 27;7(8):e016404. doi: 10.1136/bmjopen-2017-016404 (PMC5629738; doi:10.1136/bmjopen-2017-016404)
Supplement: Supplementary file 1 [file bmjopen-2017-016404supp001.pdf]

Supplementary Box S1. The search strategy of PubMed

Source: PubMed

Search on: February 28th, 2017

#1 ((("vitamin D"[Mesh] OR "cholecalciferol"[Mesh]) OR "25-hydroxyvitamin D"[Title/Abstract]) OR "25(OH)D"[Title/Abstract])

#2 ((("small for gestational age"[Title/Abstract] OR "small-for-gestation-age"[Title/Abstract]) OR "small size for gestational age"[Title/Abstract]) OR SGA [Title/Abstract])

#3 #1 AND #2

The search strategy in other databases did some adjustments on the basis of the above database.

Supplementary Table S1. Characteristics of the included studies in the present meta-analysis

Author	Latitude	The time of year data collected	Gestational age of infant at birth	The prevalence of SGA	The prevalence of vitamin D deficiency	Maternal education status	Season of blood sample
Leffelaar[15]	NA	2003.2~2004.3	40.1±1.2 weeks	9.2%	23.1%	≤5 years (17.2%), 6-10years (38.5%), ≥11 years (44.3%)	Summer (43.6%)
Burris[24]	NA	NA	39.6 weeks	4.8%	32.4%	College graduate (41.2%)	NA
Zhou[25]	23.1°N	2010.9~2011.8	NA	0.6%	18.9%	NA	NA
Choi[26]	36.0°N	2012.4~2013.9	NA	10.9%	77.3%	≤12 years(5.5%), > 12 years(94.5%)	Spring (44.5%), Summer (10.0%), Fall (39.5%), Winter (5.9%)
Ong[18]	1°22′N	NA	NA	9.1%	13.2%	Primary and secondary (30.2%), Post-secondary (35.4%), University (34.4%)	NA
Kiely[27]	52°N	2008.3~2011.2	NA	10.7%	44.0%	Secondary (61%), Tertiary (39%)	Winter (58.5%), Summer (41.5%)
Scholl[28]	NA	2001~2007	38.5	7.2%	33.7%	NA	NA
Chen[4]	31°52′N	2008.11~2010.10	NA	8.9%	38.41%	NA	Spring (36.7%), Summer (22.5%), Fall (20.6%), Winter (20.2%)
Boyle[29]	NA	2005~2008	NA	9.9%	21.5%	NA	Spring (20.5%), Summer (26.4%), Fall (23.2%), Winter (29.8%)
Berg[30]	52°22′N	2003.2~2004.3	20-42 weeks	9.1%	NA	NA	Winter (55.5%)
Gerand[16]	≥41°N(63.0%), 38~40°N(28.8%), ≤35°N(8.2%)	1959~1965	39.7±1.3 weeks	18.4%	34.8%	NA	Spring (25.9%), Summer (25.7%), Fall (24.6%), Winter (23.9%)
Miliku[31]	NA	2002.4~2006.1	35.9-42.3 weeks	5.0%	53.2%	No higher education (59.2%), Higher education (40.8%)	Spring (29.5%), Summer (22.9%), Fall (24.0%), Winter (23.6%)
Nobles[17]	NA	2007~2012	NA	9.6%	20.7%	≤High school (55.2%), > High school (44.8%)	Summer (41.4%), Winter (58.7%)

NA：not available.

Supplementary Box S2. Quality assessment of cohort studies

NEWCASTLE - OTTAWA QUALITY ASSESSMENT SCALE
COHORT STUDIES

Note: A study can be awarded a maximum of one star for each numbered item within the Selection and Outcome categories. A maximum of two stars can be given for Comparability

Selection

1) Representativeness of the exposed cohort
a) truly representative of the average _______________ (describe) in the community ❋
b) somewhat representative of the average ______________ in the community ❋
c) selected group of users eg nurses, volunteers
d) no description of the derivation of the cohort
2) Selection of the non-exposed cohort
a) drawn from the same community as the exposed cohort ❋
b) drawn from a different source
c) no description of the derivation of the non-exposed cohort
3) Ascertainment of exposure
a) secure record (eg surgical records) ❋
b) structured interview ❋
c) written self-report
d) no description
4) Demonstration that outcome of interest was not present at start of study
a) yes ❋
b) no

Comparability

1) Comparability of cohorts on the basis of the design or analysis
a) study controls for _____________ (select the most important factor) ❋
b) study controls for any additional factor ❋ (This criteria could be modified to indicate specific control for a second important factor.)

Outcome

1) Assessment of outcome
a) independent blind assessment ❋
b) record linkage ❋
c) self-report
d) no description
2) Was follow-up long enough for outcomes to occur
a) yes (select an adequate follow up period for outcome of interest) ❋
b) no
3) Adequacy of follow up of cohorts
a) complete follow up - all subjects accounted for ❋
b) subjects lost to follow up unlikely to introduce bias - small number lost - > ____ % (select an adequate %) follow up, or description provided of those lost) ❋
c) follow up rate < ____% (select an adequate %) and no description of those lost
d) no statement

Supplementary Table S2. Quality scores of included studies on vitamin D status and SGA.

Study	Selection				Comparability	Outcome			Total scores
	Representativeness of the exposed cohort	Selection of the non-exposed cohort	Ascertainment of exposure	Demonstration that outcome of interest was not present at start of study	Comparability of cohorts on the basis of design or analysis	Assessment of outcome	Was follow-up long enough for outcomes to occur	Adequacy of follow up of cohorts	
Leffelaar[14]	1	1	1	1	1	1	1	1	8
Burris[23]	1	1	1	1	1	1	1	0	7
Zhou[24]	1	1	1	1	1	1	1	1	8
Choi[25]	1	1	1	1	1	1	0	0	6
Ong[17]	1	1	1	1	2	1	1	0	8
Kiely[26]	1	0	1	1	1	1	1	0	6
Scholl[27]	1	1	1	1	2	0	1	1	8
Chen[4]	1	1	1	1	0	1	1	0	6
Boyle[28]	1	1	1	1	1	1	1	0	7
Berg[29]	1	1	1	1	1	1	1	0	7
Gerand[15]	1	1	1	1	0	1	1	0	6
Miliku[30]	1	1	1	1	1	1	1	0	7
Nobles[16]	1	1	1	1	1	1	1	1	8
