# Supplementary material for: Association between maternal vitamin D deficiency and small for gestational age: evidence from a meta-analysis of prospective cohort studies
Source: BMJ Open. 2017 Aug 27;7(8):e016404. doi: 10.1136/bmjopen-2017-016404 (PMC5629738; doi:10.1136/bmjopen-2017-016404)
Supplement: Author's manuscript [file bmjopen-2017-016404.draft_revisions.pdf]

BMJ Open

BMJ Open is committed to open peer review. As part of this commitment we make the peer review history of every article we publish publicly available.

When an article is published we post the peer reviewers' comments and the authors' responses online. We also post the versions of the paper that were used during peer review. These are the versions that the peer review comments apply to.

The versions of the paper that follow are the versions that were submitted during the peer review process. They are not the versions of record or the final published versions. They should not be cited or distributed as the published version of this manuscript.

BMJ Open is an open access journal and the full, final, typeset and author-corrected version of record of the manuscript is available on our site with no access controls, subscription charges or pay-per-view fees (http://bmjopen.bmj.com).

If you have any questions on BMJ Open's open peer review process please email info.bmjopen@bmj.com

BMJ Open

Association between maternal vitamin D deficiency and small for gestational age: evidence from a meta-analysis of prospective cohort studies

Journal:	*BMJ Open*
Manuscript ID	bmjopen-2017-016404
Article Type:	Research
Date Submitted by the Author:	11-Feb-2017
Complete List of Authors:	Chen, Yao; Anhui Medical University, School of Public Health<br>Li, Si; Anhui Medical University, School of Public Health<br>Wu, Xiaoyan<br>Zhu, Beibei; Anhui Medical University, School of Public Health<br>Tao, Fangbiao; Anhui Provincial Key Laboratory of Population Health and Aristogenics,
**Primary Subject Heading**:	Public health
Secondary Subject Heading:	Nutrition and metabolism
Keywords:	vitamin D, small for gestational age, cohort study, meta-analysis

SCHOLARONE™ Manuscripts

For peer review only

Association between maternal vitamin D deficiency and small for gestational age: evidence from a meta-analysis of prospective cohort studies

Yao Chen[1], Si Li[2], Xiaoyan Wu[1,3], Beibei Zhu[1,3], Fangbiao Tao[1,3]

[1]Department of Maternal, Child and Adolescent Health, School of Public Health, Anhui Medical University, Hefei, China

[2]Department of Epidemiology and Biostatistics, School of Public Health, Anhui Medical University, Hefei, China.

[3]Anhui Provincial Key Laboratory of Population Health and Aristogenics, Hefei, China

Correspondence to: Fangbiao Tao, Department of Maternal, Child and Adolescent Health, School of Public Health, Anhui Medical University, 81Meishan Road, Hefei, Anhui, 230032, P.R.China. Tel.: +86 551 65161169.

Email: fbtao@ahmu.edu.cn

For peer review only - http://bmjopen.bmj.com/site/about/guidelines.xhtml

ABSTRACT

Objective: To estimate whether maternal vitamin D deficiency during pregnancy is associated with small for gestational age (SGA).

Methods: A comprehensive literature search of PubMed, Cochrane Library, EMBASE and Elsevier ScienceDirect library was conducted to identify relevant articles of prospective cohort studies in English, with the last report up to February 2017. Pooled odds ratio (OR) and corresponding 95% confidence interval (95% CI) were used to evaluate the correlation in either random or fixed effects model according to between-study heterogeneity.

Results: Thirteen cohort studies are included in this meta-analysis. Maternal with circulating 25-hydroxyvitamin D [25(OH)D] deficiency experienced had an increased risk of SGA (pooled OR=1.574; 95% CI 1.124 to 2.204; P<0.01). Subgroup analysis revealed that pregnant women with a vitamin D level below 10 ng/ml (OR=2.219, 95% CI 1.480 to 3.325) and below 15 ng/ml (OR=1.532, 95% CI 1.046 to 2.246) had a significantly increased risk of SGA, however, this association didn't exist in subgroup of below 20 ng/ml (OR=1.424, 95% CI 0.828 to 2.449). When stratified according to blood sampling weeks, we found only blood sampling from the second trimester showed a positive association (OR=1.544, 95% CI 1.088 to 2.192)

Conclusion: Our meta-analysis suggests that vitamin D deficiency is associated with an increased risk of SGA and the optimal cutoff and critical timing need further investigation.

Keywords: vitamin D; small for gestational age; cohort study; meta-analysis.

Strengths and limitations of this study: This study only included prospective cohort studies, which have more advantages than case-control studies. Subgroups analysis of this study presented more thorough understanding of current evidence. Quality of each cohort study, heterogeneity test, sensitivity analysis and publication bias were conducted. Different definition of vitamin D deficiency, insufficiency or sufficiency might have influenced the result. Substantial heterogeneity existed among several outcomes.

For peer review only

INTRODUCTION

Vitamin D is fat-soluble and a steroid hormone recognized for its major role in calcium metabolism and bone health.[1] Vitamin D deficiency or insufficiency has become a global public health issue,[2] especially for pregnant women, among whom the highest deficiency rate is up to 84% according to a multiethnic population survey in Norway.[3] Several large studies have depicted the associations of maternal vitamin D deficiency with various adverse maternal and fetal outcomes[4-6] including SGA.

SGA are defined as smaller in size for their own gestational age, most commonly recognized as a weight below the 10th percentile for corresponding gestational age.[7 8] The incidence of SGA was 9.7% worldwide[9] with a growth tendency. Infants born to SGA have much higher neonatal morbidity and mortality.[10] Even worse, it might also do a lot harm to other well-beings throughout childhood to adulthood, such as neurocognitive impairment, poor school performance and short stature, as well as increased the risk of diabetes,[11] cardiovascular disease[12] and kidney disease.[13]

Although many studies have focused on the association between maternal vitamin D status and SGA, the results of these studies remain inconsistent. A prospective cohort study in Netherlands examining vitamin D concentrations in 3,730 pregnant women at 12-14 weeks of gestation shows that infants born to mothers with vitamin D deficiency had an increased risk of SGA compared with adequate vitamin D levels.[14] Subsequently, Gernand et al.[15] reported that the vitamin D levels below 15 ng/ml group had significantly higher risk of SGA. However, some other studies demonstrated no association between vitamin D status and SGA.[16 17]

Given the blurred picture of this issue, we attempted to summarize current best quality of evidences and conduct a meta-analysis of prospective cohort studies to answer whether vitamin D deficiency in pregnant women is associated with SGA.

MATERIALS AND METHODS

Data sources, search strategy, and selection criteria

A systematic literature search was performed using the PubMed, Elsevier

For peer review only - http://bmjopen.bmj.com/site/about/guidelines.xhtml

ScienceDirect and Cochrane Library databases to find out all relevant publications until February 2017. No restrictions were placed on maternal age, study design or language. The following main search terms were used: ('vitamin D' or 'cholecalciferol' or '25-hydroxyvitamin D' or '25(OH)D' and 'SGA' or 'small for gestational age' or 'small-for-gestation-age' or 'small size for gestational age')

Selection Criteria

We first screened the titles and abstracts of all the articles to identify the possible eligible studies, and then read the full articles to include eligible studies. The studies fit into the meta-analysis were selected according to the following criteria: 1) cohort studies evaluated the association between vitamin D status and risk of SGA; 2) studies with data in the form of effect estimate [odds ratio (OR) or risk ratio (RR)] and corresponding 95% confidence interval (CI) or reported data to calculate them; 3) maternal blood samples were taken for assessing 25(OH)D before or at delivery; 4) studies with pregnant women suffering from other metabolic disease were excluded.

Data extraction and quality evaluation

Two investigators respectively reviewed all abstracts for related studies, and read full texts of eligible literatures, extracted data using a standardized form and assessed study quality. Disagreements were resolved by discussion and by consulting a third investigator. The following data were collected from each study: the first author name, nation, publish year, the average age and pre-pregnancy body mass index (BMI) of study populations, current gestational week of blood sampling, assay methods of serum/plasma vitamin D levels measured and sample size. If original important data were unavailable, we contacted the corresponding author by e-mail to obtain further details. Finally, we assessed the eligible studies based on Newcastle-Ottawa Scale (NOS) system. This scale ranging from 0 to 9 contains nine items (1 point for each) in three parts: selection (four items), comparability (two items)

For peer review only - http://bmjopen.bmj.com/site/about/guidelines.xhtml

and exposure or outcomes (three items). Scores ranging from 0-3 were deemed to poor quality, scores ranging from 4 to 6 were deemed to moderate quality and scores surpassing 7 were deemed to high quality.

Statistical Analysis

The data extracted from eligible studies were in the form of effect estimate [odds ratio (OR) or risk ratio (RR)] and corresponding 95% confidence interval (CI). Due to the low level of the morbidity of SGA, the value of OR was approximately equal to RR.[18] Meta-analysis was performed using STATA package version 12.0 (Stata Corporation, College Station, TX, USA). The ORs and 95% CIs for normal vitamin D levels versus deficient vitamin D levels from each study were combined to calculate the estimated pooled OR, 95% CI and *P* value. Q-statistic test and the I-square (I^2) test were used to estimate the heterogeneity among different studies.[19] The fixed-effects model was used for meta-analysis when I^2 was under 50% and *P* value surpassed 0.05, otherwise, the random-effects model was used.[20] To explore the sources of heterogeneity and the various results of subgroups, Subgroup analysis was carried out based on status of ethnicity, cut-off values, study quality, adjustment of critical confounders, sample size, and current gestation of blood sampling. A sensitivity analysis was conducted to determine the stability and reliability of the results by leave one out at a time and checking the consistency of the overall effect estimate. Funnel plots were used to qualitatively assess the publication bias, and Egger's and Begg's tests were also used to quantitatively assess publication bias.[21 22]

RESULTS

Description of included studies

A total of 1734 literatures were identified for initial review using search strategies as described. 1537 literatures were removed according to the inclusion and exclusion criteria (figure 1). Because of the unavailability of data, 4 studies were excluded.

For peer review only - http://bmjopen.bmj.com/site/about/guidelines.xhtml

Finally, 13 cohort studies[4 14-17 23-30] were included in the meta-analysis, including 28285 pregnant women.

Characteristics and methodological quality of 13 studies are presented in table 1: The population sources of studies were Caucasian (9 studies) and Asian (4 studies). The average age of the pregnant women of those studies was < 30 years old (4 studies) and >30 years old (5 studies). Of the 13 studies, the average pre-pregnancy BMI of 7 studies were below 25 kg/m^2 and 3 studies were above 25 kg/m^2. 10 studies have adjusted for confounders and 3 studies have not. 6 studies adopted blood during first trimester, 5 studies were second trimester. Furthermore, 7 studies assessed the serum/plasma levels of vitamin D by the way of LS-MS, 6 studies used other methods. Finally, the degrees of NOS score were high levels (9 studies) and low levels (4 studies).

Meta-analysis results

The Coherence Q test showed the existence of heterogeneity in the meta-analysis (I^2=84.1%; *P*<0.001), so the random-effects model was applied. The overall results showed that maternal vitamin D deficiency during pregnancy is significantly associated with an increased risk of SGA (pooled OR=1.574; 95% CI 1.124 to 2.204; *P*<0.01) and the forest plot showed the details (figure 2).

Subgroup analysis

Due to the existence of heterogeneity, subgroup analysis was carried out to explore the possible sources of heterogeneity in the meta-analysis (table 2). The subgroups were based on status of ethnicity, cut-off values of vitamin D levels, sample size, NOS score levels, whether adjusted for critical confounders and gestational week of blood sampling. The pregnant women in Caucasian, vitamin D deficiency markedly increased the risk of SGA. Moreover, maternal vitamin D deficiency during pregnancy was significantly associated with SGA in studies with high study quality, the similar results were also observed in studies with blood sampling during second

For peer review only - http://bmjopen.bmj.com/site/about/guidelines.xhtml

trimester and the heterogeneity observably reduced (pooled OR=1.544; 95% CI 1.088 to 2.192; I^2=48.2%; P=0.102). Furthermore, subgroup analysis according to whether adjusted for critical confounders, the NOS score levels and sample size of study all showed significant results, and the studies with the cut-off values of vitamin D status <15 ng/ml or <10 ng/ml showed markedly relevance between SGA and vitamin D, noteworthy, the heterogeneity significant decline (pooled OR=1.532; 95% CI 1.04 to 2.246; I^2=73.2%; P=0.054 vs. pooled OR=2.219; 95% CI 1.046 to 2.246; I^2=0; P=0.446).

Sensitivity analysis and publication bias

To evaluate the stability of our results, sensitivity analysis was carried out and the results revealed that the OR and 95% CI were stable when any one study was excluded using random-effect methods (table 3). There was also no publication bias after carrying out Begg's test (P=0.760) and Egger's regression test (P=0.852), the funnel plot showed the details (figure 3).

DISCUSSION

The prevalence of vitamin D deficiency during pregnancy and its association with risk of SGA caught more and more attentions. Current meta-analysis of prospective cohort studies suggests that vitamin D deficiency is significantly associated with an increased risk of SGA. No publication bias was detected and sensitive analysis showed no single study dramatically influences the results, which indicated that the results of our meta-analysis are stable and reliable.

Our study is in line with several previous studies. A previous meta-analysis showed that low maternal vitamin D levels during pregnancy may be associated with an increased risk of SGA, gestational diabetes mellitus, preterm birth.[5] Similarly, another vital meta-analysis also suggested that vitamin D insufficiency is associated with an increased risk of SGA, preeclampsia, and bacterial vaginosis.[6] However, those studies included both case-control and prospective cohort studies and did not include latest

For peer review only

For peer review only - http://bmjopen.bmj.com/site/about/guidelines.xhtml

published cohort studies, and did not evaluate the association in specific subgroup analysis. Moreover, the cut-off values for the vitamin D status differed between different studies. Thus, we conducted this meta-analysis to provide stronger evidence for the association between vitamin D and SGA.

Heterogeneity test (the Coherence Q test) showed significant heterogeneity existed among studies. We explored the potential influential factors for the results by performing a subgroup analysis. And we obtained positive results from the following subgroups: Caucasian population, blood sampling from the second trimester, sample size exceeded 1000, the studies of high quality and adjusted potential confounder factors. A case-control study conducted in UK measured the 25(OH)D levels of maternal at 11-13 weeks of gestation and showed that serum 25(OH)D levels were decreased in Caucasian women that deliver SGA, but was not observed in African women,[31] which was consistent with our results. Furthermore, we found pregnant women with a vitamin D level below 10 ng/ml (OR=2.219, 95% CI 1.480 to 3.325) and below 15 ng/ml (OR=1.532, 95% CI 1.046 to 2.246) had a significantly increased risk of SGA, however, such association was not obvious in the subgroup of 25(OH)D <20 ng/ml. Therefore, the cut-offs of vitamin D deficiency needs further exploration

The underlying mechanism of vitamin D deficiency increases risk of SGA is not entirely clear but might be explained by the inflammatory response. Vitamin D deficiency can increase the levels of proinflammatory cytokines, leading to oxidative stress. Lower 25(OH)D status is associated with increased vascular endothelial cell expression of nuclear factor κB (NFκB) and interleukin 6 (IL-6), and with decreased of vitamin D receptor (VDR) and 1-α hydroxylase.[32] A study reported that the levels of pro-inflammatory cytokines in cord blood of SGA are significantly higher than that in normal-born infants.[33] Mullins et al.[34] show that the offspring pregnant women of SGA contained higher tumor necrosis factor (TNF-α) than normal offspring of pregnant women, and as an important inflammatory factor, TNF-α inhibited placental hormone synthesis and stimulated calcitriol catabolism by regulating enzymes.[35] Vitamin D may also play an important role in innate and adaptive immunity by

For peer review only - http://bmjopen.bmj.com/site/about/guidelines.xhtml

inhibiting the pathway of decidual NFκB to reduce the inflammatory response, since NFκB is a main transcription factor of inflammatory mediators.[36]

Maternal vitamin D deficiency is prevalent, the extent of which can be influenced by many variables including ethnicity, region, skin pigmentation, sun exposure, season, age, vitamin D supplementation and others.[37] The American Association of Endocrinology recommended that pregnant women require at least 600 IU/d of vitamin D and confirmed that at least 1500-2000 IU/d of vitamin D may be needed to keep a blood level of vitamin D above 30 ng/ml.[38] However, the recommendations of pregnant women vitamin D supplementation is scanty. At present, vitamin D supplementation during pregnancy has been suggested as an intervention to prevent adverse pregnancy outcomes.[39] A randomized controlled trial reported that maternal vitamin D supplementation of 2000 and 4000 IU/d appeared safe during pregnancy, and the most effective in optimizing serum vitamin D concentrations in mothers and their infants was 4000 IU/d,[40] this result was consistent with another randomized controlled trial in Pakistan.[41] Low vitamin D levels during pregnancy could increase the risk of SGA, however, vitamin D supplementation did not significantly reduce the risk of SGA (OR=0.78, 95% CI 0.50 to 1.21).[42] Therefore, we need larger randomized controlled trials to assess the value of these interventions in the future, which has a significant impact on the guidance of the perinatal period care.

Our study has several strengthens. First, to provide more reliable evidence, we only included prospective cohort studies, which have more advantages than case-control studies as we all familiar with. Second, no publication bias existing, indicating that the included results may be unbiased and credible. Third, subgroups analysis of our study presented more thorough understanding of current evidence. Several limitations should also be acknowledged. First, the association between maternal vitamin D status and SGA risk could be affected by confounding factors such as pre-pregnancy BMI, age, education, race and exposure sunlight, however, not all studies are control these confounding factors in our meta-analysis. Second, different definition of vitamin D deficiency, insufficiency or sufficiency might have influenced the result. Third,

For peer review only

pooled data without detail individual information were used to performed meta-analysis, which restricted us to get more comprehensive results.

CONCLUSIONS

The present study indicates that low vitamin D levels is associated with an increased risk of SGA. Further confirmation of these findings in larger sample size studies is required. The role of vitamin D in the pathogenesis of SGA should be emphasized. As well, early screening for vitamin D deficiency of pregnant women may be necessary under the background of this study.

Contributors FT contributed to study design; SL, XW and BZ contributed analysis tools and methods; YC analyzed the data and drafted the manuscript; BZ and FT revised the manuscript. All authors read and approved the final version of the manuscript.

Funding This work is grateful for the financial support offered by the National Natural Science Foundation of China (81330068, 81573168)

Competing interests None declared.

For peer review only - http://bmjopen.bmj.com/site/about/guidelines.xhtml

For peer review only

REFERENCES

1. Finberg L. Vitamin D deficiency and rickets. *J Pediatr Endocrinol Metab* 2006; 19:203.
2. Palacios C, Gonzalez L. Is vitamin D deficiency a major global public health problem? *J Steroid Biochem Mol Biol* 2014; 144 Pt A:138-145.
3. Eggemoen AR, Falk RS, Knutsen KV, et al. Vitamin D deficiency and supplementation in pregnancy in a multiethnic population-based cohort. *BMC pregnancy and childbirth* 2016; 16:7.
4. Chen YH, Fu L, Hao JH, et al. Maternal vitamin D deficiency during pregnancy elevates the risks of small for gestational age and low birth weight infants in Chinese population. *J Clin Endocrinol Metab* 2015; 100:1912-1919.
5. Wei SQ, Qi HP, Luo ZC, et al. Maternal vitamin D status and adverse pregnancy outcomes: a systematic review and meta-analysis. *J Matern Fetal Neonatal Med* 2013; 26:889-899.
6. Aghajafari F, Nagulesapillai T, Ronksley PE, et al. Association between maternal serum 25-hydroxyvitamin D level and pregnancy and neonatal outcomes: systematic review and meta-analysis of observational studies. *BMJ* 2013; 346:f1169.
7. Mikolajczyk RT, Zhang J, Betran AP, et al. A global reference for fetal-weight and birthweight percentiles. *Lancet* 2011; 377:1855-1861.
8. Hoftiezer L, Hukkelhoven CW, Hogeveen M, et al. Defining small-for-gestational-age: prescriptive versus descriptive birthweight standards. *Eur J Pediatr* 2016; 175:1047-1057.
9. Ding G, Tian Y, Zhang Y, et al. Application of a global reference for fetal-weight and birthweight percentiles in predicting infant mortality. *BJOG* 2013; 120(13):1613-1621.
10. Christian P, Murray-Kolb LE, Tielsch JM, et al. Associations between preterm birth, small-for-gestational age, and neonatal morbidity and cognitive function among school-age children in Nepal. *BMC pediatrics* 2014; 14:58.
11. de Zegher F, Sebastiani G, Diaz M, et al. Body composition and circulating high-molecular-weight adiponectin and IGF-I in infants born small for gestational age: breast- versus formula-feeding. *Diabetes* 2012; 61:1969-1973.
12. Wolfenstetter A, Simonetti GD, Poschl J, et al. Altered cardiovascular rhythmicity in children born small for gestational age. *Hypertension* 2012; 60:865-870.
13. Aly H, Davies J, El-Dib M, et al. Renal function is impaired in small for gestational age premature infants. *J Matern Fetal Neonatal Med* 2013; 26:388-391.
14. Leffelaar ER, Vrijkotte TG, van Eijsden M. Maternal early pregnancy vitamin D status in relation to fetal and neonatal growth: results of the multi-ethnic Amsterdam Born Children and their Development cohort. *Br J Nutr* 2010; 104:108-117.
15. Gernand AD, Simhan HN, Klebanoff MA, et al. Maternal serum 25-hydroxyvitamin D and measures of newborn and placental weight in a U.S. multicenter cohort study. *J Clin Endocrinol Metab* 2013; 98:398-404.
16. Nobles CJ, Markenson G, Chasan-Taber L. Early pregnancy vitamin D status and risk for adverse maternal and infant outcomes in a bi-ethnic cohort: the Behaviors Affecting Baby and You (B.A.B.Y.) Study. *Br J Nutr* 2015; 114:2116-2128.
17. Ong YL, Quah PL, Tint MT, *et al*. The association of maternal vitamin D status with infant birth outcomes, postnatal growth and adiposity in the first 2 years of life in a multi-ethnic Asian population: the Growing Up in Singapore Towards healthy Outcomes (GUSTO) cohort study.

For peer review only

For peer review only - http://bmjopen.bmj.com/site/about/guidelines.xhtml

Br J Nutr 2016, 116:621-631.

18. Gagne JJ, Power MC. Anti-inflammatory drugs and risk of Parkinson disease: a meta-analysis. *Neurology* 2010; 74:995-1002.
19. Takkouche B, Cadarso-Suarez C, Spiegelman D. Evaluation of old and new tests of heterogeneity in epidemiologic meta-analysis. *Am J Epidemiol* 1999; 150:206-215.
20. Higgins JP, Thompson SG, Deeks JJ, et al. Measuring inconsistency in meta-analyses. *BMJ* 2003; 327:557-560.
21. Egger M, Davey Smith G, Schneider M, et al. Bias in meta-analysis detected by a simple, graphical test. *BMJ* 1997; 315:629-634.
22. Begg CB, Mazumdar M. Operating characteristics of a rank correlation test for publication bias. *Biometrics* 1994; 50:1088-1101.
23. Burris HH, Rifas-Shiman SL, Camargo CA, et al. Plasma 25-hydroxyvitamin D during pregnancy and small-for-gestational age in black and white infants. *Ann Epidemiol* 2012; 22:581-586.
24. Zhou J, Su L, Liu M, et al. Associations between 25-hydroxyvitamin D levels and pregnancy outcomes: a prospective observational study in southern China. *Eur J Clin Nutr* 2014; 68:925-930.
25. Choi R, Kim S, Yoo H, et al. High prevalence of vitamin D deficiency in pregnant Korean women: the first trimester and the winter season as risk factors for vitamin D deficiency. *Nutrients* 2015; 7:3427-3448.
26. Kiely ME, Zhang JY, Kinsella M, et al. Vitamin D status is associated with uteroplacental dysfunction indicated by pre-eclampsia and small-for-gestational-age birth in a large prospective pregnancy cohort in Ireland with low vitamin D status. *Am J Clin Nutr* 2016; 104:354-361.
27. Scholl TO, Chen X, Stein TP. Maternal calcium metabolic stress and fetal growth. *Am J Clin Nutr* 2014; 99:918-925.
28. Boyle VT, Thorstensen EB, Mourath D, et al. The relationship between 25-hydroxyvitamin D concentration in early pregnancy and pregnancy outcomes in a large, prospective cohort. *Br J Nutr* 2016; 116:1409-1415.
29. van den Berg G, van Eijsden M, Vrijkotte TG, et al. Suboptimal maternal vitamin D status and low education level as determinants of small-for-gestational-age birth weight. *Eur J Nutr* 2013; 52:273-279.
30. Miliku K, Vinkhuyzen A, Blanken LM, *et al*. Maternal vitamin D concentrations during pregnancy, fetal growth patterns, and risks of adverse birth outcomes. *Am J Clin Nutr* 2016; 103:1514-1522.
31. Ertl R, Yu CK, Samaha R, et al. Maternal serum vitamin D at 11-13 weeks in pregnancies delivering small for gestational age neonates. *Fetal Diagn Ther* 2012; 31:103-108.
32. Jablonski KL, Chonchol M, Pierce GL, et al. 25-Hydroxyvitamin D deficiency is associated with inflammation-linked vascular endothelial dysfunction in middle-aged and older adults. *Hypertension* 2011; 57:63-69.
33. Lausten-Thomsen U, Olsen M, Greisen G, et al. Inflammatory markers in umbilical cord blood from small-for-gestational-age newborns. *Fetal Pediatr Pathol* 2014; 33:114-118.
34. Mullins E, Prior T, Roberts I, et al. Changes in the maternal cytokine profile in pregnancies complicated by fetal growth restriction. *Am J Reprod Immunol* 2012; 68:1-7.
35. Diaz L, Noyola-Martinez N, Barrera D, et al. Calcitriol inhibits TNF-alpha-induced

For peer review only - http://bmjopen.bmj.com/site/about/guidelines.xhtml

inflammatory cytokines in human trophoblasts. *J Reprod Immunol* 2009; 81:17-24.

36. Cohen-Lahav M, Shany S, Tobvin D, et al. Vitamin D decreases NFkappaB activity by increasing IkappaBalpha levels. *Nephrol Dial Transplant* 2006; 21:889-897.

37. Bodnar LM, Simhan HN. Vitamin D may be a link to black-white disparities in adverse birth outcomes. *Obstet Gynecol Surv* 2010; 65:273-284.

38. Holick MF, Binkley NC, Bischoff-Ferrari HA, et al. Evaluation, treatment, and prevention of vitamin D deficiency: an Endocrine Society clinical practice guideline. *J Clin Endocrinol Metab* 2011; 96:1911-1930.

39. De-Regil LM, Palacios C, Lombardo LK, et al. Vitamin D supplementation for women during pregnancy. *Sao Paulo Med* 2016; 134:274-275.

40. Dawodu A, Saadi HF, Bekdache G, et al. Randomized controlled trial (RCT) of vitamin D supplementation in pregnancy in a population with endemic vitamin D deficiency. *J Clin Endocrinol Metab* 2013; 98:2337-2346.

41. Hossain N, Kanani FH, Ramzan S, et al. Obstetric and neonatal outcomes of maternal vitamin D supplementation: results of an open-label, randomized controlled trial of antenatal vitamin D supplementation in Pakistani women. *J Clin Endocrinol Metab* 2014; 99:2448-2455.

42. Perez-Lopez FR, Pasupuleti V, Mezones-Holguin E, et al. Effect of vitamin D supplementation during pregnancy on maternal and neonatal outcomes: a systematic review and meta-analysis of randomized controlled trials. *Fertil Steril* 2015; 103:1278-1288 e1274

For peer review only

For peer review only - http://bmjopen.bmj.com/site/about/guidelines.xhtml

Table 1. Characteristics of the included studies in the present meta-analysis

Author	Region	Year	Age at baseline	Pre-pregnancy BMI (kg/m^2)	Gestational week of blood sampling	Measurement of vitamin D	NOS Score	Sample size
Leffelaar[14]	Netherlands	2010	NA	NA	12-14 weeks	enzyme immunoassay	8	3730
Burris[23]	USA	2012	32.5	24.8	26-28 weeks	CLIA and RIA	7	1133
Zhou[24]	China	2014	29.5	20.3	16-20 weeks	ECLIA	8	1923
Choi[25]	Korea	2015	32.0	20.2	first or second or third trimester	LC-MS/MS	6	220
Ong[17]	Singapore	2016	30.5	26.1	26-28 weeks	LC-MS/MS	8	910
Kiely[26]	Ireland	2016	30.5	24.9	14-16 weeks	LC-MS/MS	6	1768
Scholl[27]	USA	2014	22.8	26	13.8±5.6 weeks	RIA	8	1045
Chen[4]	China	2015	27.5	NA	first or second or third trimester	RIA	6	3658
Boyle[28]	New Zealand	2016	30.3	24.8	15 weeks	LC-MS/MS	7	2065
Berg[29]	Netherlands	2013	NA	NA	12.9 weeks	enzyme immunoassay	7	2274
Gerand[15]	USA	2013	NA	22.3	20.6 weeks	LC-MS/MS	6	2146
Miliku[30]	Netherlands	2016	29.7	23.7	20.3 weeks	LC-MS/MS	7	7176
Nobles[16]	USA	2015	NA	>25	15.2±4.7 weeks	LC-MS/MS	8	237

CLIA: chemiluminescence immunoassay; RIA: radioimmunoassay; ECLIA: electrochemiluminescence immunoassay; LC-MS/MS: liquid chromatography-tandem mass spectrometry; NA: not available.

For peer review only

Table 2. Subgroup analysis of the association between maternal Vitamin D deficiency and SGA

Stratification group	N	P Value for OR	OR (95% CI)	Heterogeneity test: I-square (%)	Heterogeneity test: P Value
Ethnicity					
Caucasian	9	0.001	1.433 (1.150, 1.785)	57.2	0.016
Asian	4	0.475	1.655 (0.416, 6.587)	92.3	<0.001
Study quality (NOS)					
High	9	<0.001	1.542 (1.230, 1.934)	36.0	0.130
Low	4	0.440	1.441 (0.570, 3.641)	95.2	<0.001
Gestation of blood sampling					
first trimester	6	0.058	1.320 (0.991,1.760)	59.5	0.030
second trimester	5	0.015	1.544 (1.088, 2.192)	48.2	0.102
Cut-off values					
<10 ng/ml	2	0.001	2.219 (1.480, 3.325)	0	0.446
<15 ng/ml	2	0.029	1.532(1.046, 2.246)	73.2	0.054
<20 ng/ml	9	0.201	1.424 (0.828, 2.449)	88.2	<0.001
Sample size					
> 1000	10	0.003	1.745(1.201, 2.536)	86.7	<0.001
< 1000	3	0.946	0.975(0.476, 1.999)	45.5	0.160
Adjust for critical confounders					
yes	10	0.018	1.681 (1.094, 2.584)	86.3	< 0.001
no	3	0.051	1.224 (0.999, 1.500)	0	0.395

For peer review only

Table 3. Sensitivity analyses of the association between vitamin D deficiency and SGA

Study omitted	*OR* (95% CI)	*P* value	I-square (%)	*P* value
Leffelaar[14]	1.543 (1.058, 2.250)	0.024	85.1	< 0.001
Burris[23]	1.511 (1.068, 2.138)	0.020	85.1	< 0.001
Zhou[24]	1.557 (1.105, 2.195)	0.011	85.4	< 0.001
Choi[25]	1.679 (1.196, 2.357)	0.003	84.4	< 0.001
Ong[17]	1.638 (1.147, 2.339)	0.007	85.0	< 0.001
Kiely[26]	1.672 (1.175, 2.377)	0.004	83.3	< 0.001
Scholl[27]	1.654 (1.159, 2.361)	0.006	84.5	< 0.001
Chen[4]	1.354 (1.094, 1.676)	0.005	54.5	0.012
Boyle[28]	1.601 (1.102, 2.324)	0.013	85.3	< 0.001
Berg[29]	1.574 (1.086, 2.281)	0.017	85.4	< 0.001
Gerand[15]	1.608 (1.084, 2.387)	0.018	84.7	< 0.001
Miliku[30]	1.532 (1.063, 2.207)	0.022	85.1	< 0.001

For peer review only

For peer review only - http://bmjopen.bmj.com/site/about/guidelines.xhtml

Nobles[16]	1.550 (1.094, 2.196)	0.014	85.4	< 0.001

For peer review only

For peer review only - http://bmjopen.bmj.com/site/about/guidelines.xhtml

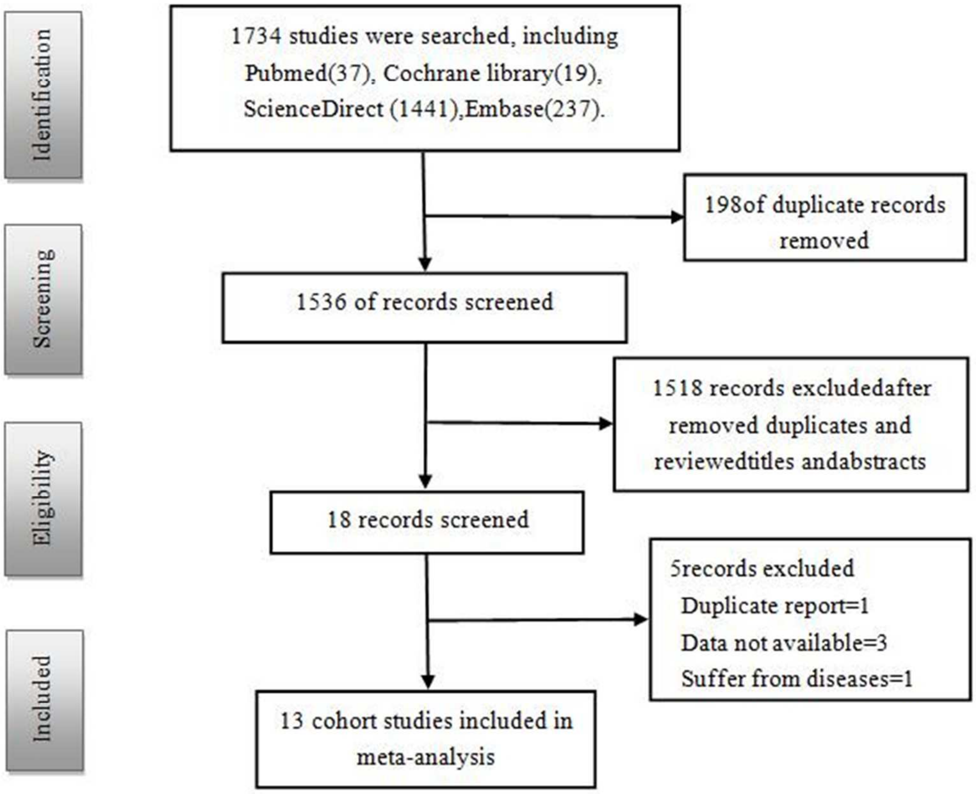

Figure 1. Flowchart of the literature search and trial selection process

162x134mm (96 x 96 DPI)

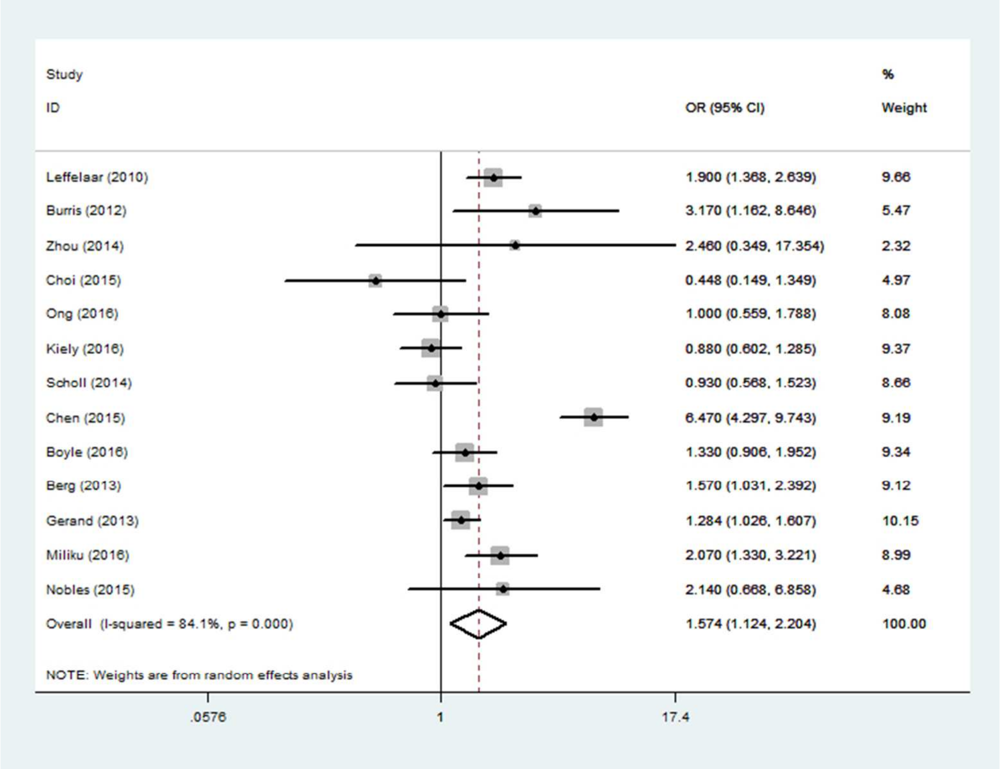

Figure 2. Forest plots of summary crude odds ratios of the association between vitamin D deficiency and SGA

259x200mm (72 x 72 DPI)

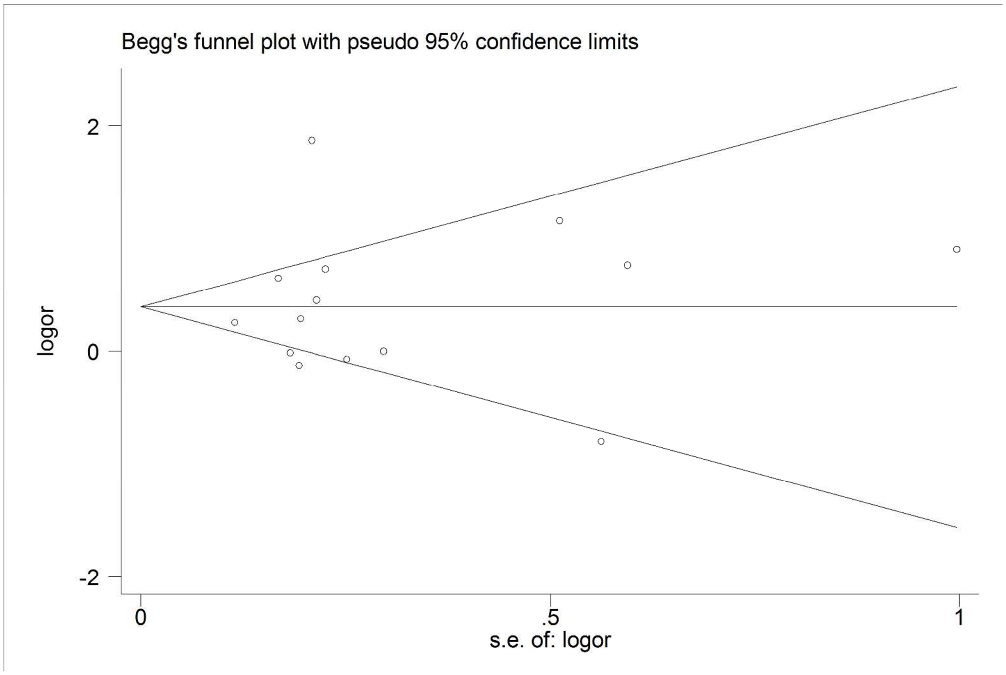

Figure 3. Funnel plot for small for gestational age. Log OR of the individual studies plotted against the standard error of log OR.

635x423mm (144 x 144 DPI)

view only

Section and topic	Item No	Checklist item	Reported on page No
Title:			
Identification	1a	Identify the report as a protocol of a systematic review	1
Update	1b	If the protocol is for an update of a previous systematic review, identify as such	
Registration	2	If registered, provide the name of the registry (such as PROSPERO) and registration number	
Authors:			
Contact	3a	Provide name, institutional affiliation, e-mail address of all protocol authors; provide physical mailing address of corresponding author	1
Contributions	3b	Describe contributions of protocol authors and identify the guarantor of the review	10
Amendments	4	If the protocol represents an amendment of a previously completed or published protocol, identify as such and list changes; otherwise, state plan for documenting important protocol amendments	
Support:			
Sources	5a	Indicate sources of financial or other support for the review	10
Sponsor	5b	Provide name for the review funder and/or sponsor	
Role of sponsor or funder	5c	Describe roles of funder(s), sponsor(s), and/or institution(s), if any, in developing the protocol	
**INTRODUCTION**			
Rationale	6	Describe the rationale for the review in the context of what is already known	3
Objectives	7	Provide an explicit statement of the question(s) the review will address with reference to participants, interventions, comparators, and outcomes (PICO)	3
**METHODS**			
Eligibility criteria	8	Specify the study characteristics (such as PICO, study design, setting, time frame) and report characteristics (such as years considered, language, publication status) to be used as criteria for eligibility for the review	4
Information sources	9	Describe all intended information sources (such as electronic databases, contact with study authors, trial registers or other grey literature sources) with planned dates of coverage	3
Search strategy	10	Present draft of search strategy to be used for at least one electronic database, including planned limits, such that it could be repeated	3

For peer review only

Study records:			
Data management	11a	Describe the mechanism(s) that will be used to manage records and data throughout the review	8
Selection process	11b	State the process that will be used for selecting studies (such as two independent reviewers) through each phase of the review (that is, screening, eligibility and inclusion in meta-analysis)	4,18
Data collection process	11c	Describe planned method of extracting data from reports (such as piloting forms, done independently, in duplicate), any processes for obtaining and confirming data from investigators	4,18
Data items	12	List and define all variables for which data will be sought (such as PICO items, funding sources), any pre-planned data assumptions and simplifications	14
Outcomes and prioritization	13	List and define all outcomes for which data will be sought, including prioritization of main and additional outcomes, with rationale	14-16
Risk of bias in individual studies	14	Describe anticipated methods for assessing risk of bias of individual studies, including whether this will be done at the outcome or study level, or both; state how this information will be used in data synthesis	16
Data synthesis	15a	Describe criteria under which study data will be quantitatively synthesised	5
	15b	If data are appropriate for quantitative synthesis, describe planned summary measures, methods of handling data and methods of combining data from studies, including any planned exploration of consistency (such as I^2, Kendall's τ)	5
	15c	Describe any proposed additional analyses (such as sensitivity or subgroup analyses, meta-regression)	15
	15d	If quantitative synthesis is not appropriate, describe the type of summary planned	5
Meta-bias(es)	16	Specify any planned assessment of meta-bias(es) (such as publication bias across studies, selective reporting within studies)	7
Confidence in cumulative evidence	17	Describe how the strength of the body of evidence will be assessed (such as GRADE)	7,9,16,20

For peer review only

Association between maternal vitamin D deficiency and small for gestational age: evidence from a meta-analysis of prospective cohort studies

Journal:	*BMJ Open*
Manuscript ID	bmjopen-2017-016404.R1
Article Type:	Research
Date Submitted by the Author:	02-May-2017
Complete List of Authors:	Chen, Yao; Anhui Medical University, School of Public Health<br>Zhu, Beibei; Anhui Medical University, School of Public Health<br>Wu, Xiaoyan<br>Li, Si; Anhui Medical University, School of Public Health<br>Tao, Fangbiao; Anhui Provincial Key Laboratory of Population Health and Aristogenics,
**Primary Subject Heading**:	Public health
Secondary Subject Heading:	Nutrition and metabolism
Keywords:	vitamin D, small for gestational age, cohort study, meta-analysis

SCHOLARONE™
Manuscripts

Association between maternal vitamin D deficiency and small for gestational age: evidence from a meta-analysis of prospective cohort studies

Yao Chen[1], Beibei Zhu[1,2], Xiaoyan Wu[1,2], Si Li[3], Fangbiao Tao[1,2]

[1]Department of Maternal, Child and Adolescent Health, School of Public Health, Anhui Medical University, Hefei, China

[2]Anhui Provincial Key Laboratory of Population Health and Aristogenics, Hefei, China

[3]Department of Epidemiology and Biostatistics, School of Public Health, Anhui Medical University, Hefei, China.

Correspondence to: Fangbiao Tao, Department of Maternal, Child and Adolescent Health, School of Public Health, Anhui Medical University, 81Meishan Road, Hefei, Anhui, 230032, P.R.China. Tel.: +86 551 65161169.

Email: taofangbiao@126.com

For peer review only

ABSTRACT

Objective: To estimate whether maternal vitamin D deficiency during pregnancy is associated with small for gestational age (SGA).

Methods: A comprehensive literature search of PubMed, Cochrane Library, EMBASE and Elsevier ScienceDirect library was conducted to identify relevant articles of prospective cohort studies in English, with the last report up to February 2017. Pooled odds ratio (*OR*) and corresponding 95% confidence interval (*95% CI*) were used to evaluate the correlation in random-effects model.

Results: Totally 13 cohort studies were included in this meta-analysis containing 28285 individuals from 7 countries. Pooled overall *ORs* for babies with SGA were 1.588 (*95% CI* 1.138 to 2.216; $P<0.01$) for women with vitamin D deficiency. In addition, the prevalence of vitamin D deficiency during pregnancy varied from 13.2% to 77.3%. Subgroup analyses showed that there were no significant differences in the association between vitamin D deficiency and SGA based on study quality, gestation of blood sampling, cut-off values, sample size, adjust for critical confounders and measurement of vitamin D.

Conclusion: The current meta-analysis suggests that vitamin D deficiency is associated with increased risk of SGA.

Keywords: vitamin D; small for gestational age; cohort study; meta-analysis.

Strengths and limitations of this study: 1) To our knowledge, this was the first systemic review only included prospective cohort studies evaluating the association between vitamin D and SGA. 2) Subgroups analysis of this study presented more thorough understanding of current evidence. 3) Quality of each cohort study, heterogeneity test, sensitivity analysis and publication bias were conducted. 4) Different definition of vitamin D deficiency, insufficiency or sufficiency might have influenced the result. 5) Substantial heterogeneity existed among several outcomes.

For peer review only - http://bmjopen.bmj.com/site/about/guidelines.xhtml

INTRODUCTION

Vitamin D is fat-soluble and a steroid hormone recognized for its major role in calcium metabolism and bone health.[1] Vitamin D deficiency or insufficiency has become a global public health issue,[2] especially for pregnant women, among whom the highest deficiency rate is up to 84% according to a multiethnic population survey in Norway.[3] Several large population size studies have depicted the associations of maternal vitamin D deficiency with various adverse maternal and fetal outcomes[4-6] including SGA.

SGA are defined as smaller in size for their own gestational age, most commonly recognized as a weight below the 10th percentile for corresponding gestational age.[7 8] The incidence of SGA was 9.7% worldwide[9] with a growth tendency. Infants born to SGA have much higher neonatal morbidity and mortality.[10] Katz J et al.[11] have showed that pooled RRs for infants who were SGA were 1.83 for neonatal mortality and 1.90 for post-neonatal morbidity. In addition, it might be strongly related to adverse health outcomes in adult life, such as neurocognitive impairment, poor school performance and short stature, as well as increased the risk of diabetes,[12] cardiovascular disease[13] and kidney disease.[14]

Although many studies have focused on the association between maternal vitamin D status and SGA, the results of these studies remain inconsistent. A prospective cohort study in Netherlands examined vitamin D concentrations in 3,730 pregnant women at 12-14 weeks of gestation shows that infants born to mothers with vitamin D deficiency had an increased risk of SGA compared with adequate vitamin D levels.[15] Subsequently, Gernand et al.[16] reported that the vitamin D levels below 15 ng/ml group had significantly higher risk of SGA. However, some other studies demonstrated no association between vitamin D status and SGA.[17 18]

Given the blurred picture of this issue, we attempted to summarize current best quality of evidences and conduct a meta-analysis of prospective cohort studies to answer whether vitamin D deficiency in pregnant women is associated with SGA.

For peer review only - http://bmjopen.bmj.com/site/about/guidelines.xhtml

MATERIALS AND METHODS

Data sources, search strategy, and selection criteria

A systematic literature search was performed using the PubMed, Elsevier ScienceDirect, Cochrane Library and Embase databases to find out all relevant publications until February 2017. No restrictions were placed on maternal age, study design or language. The following keywords were used: 'vitamin D' or 'cholecalciferol' or '25-hydroxyvitamin D' or '25(OH)D' in combined with 'SGA' or 'small for gestational age' or 'small-for-gestation-age' or 'small size for gestational age'(PubMed, for example, specific search strategy see supplementary box S1)

Selection Criteria

We first screened the titles and abstracts of all the articles to identify the possible eligible studies, and then read the full articles to include eligible studies. The studies fit into the meta-analysis were selected according to the following inclusion criteria: 1) the population of study was maternal without pre-chronic disease; 2) the study included maternal with singleton gestation; 3) the outcome was SGA and the control group included maternal without SGA, the exposure was 'vitamin D deficiency' [25(OH)D<20ng/ml]; 4) studies with data in the form of effect estimate [odds ratio (*OR*) or risk ratio (*RR*)] and corresponding 95% confidence interval (*CI*) or reported data to calculate them; 5) maternal blood samples were taken for assessing 25(OH)D during pregnancy; 6) the study design was cohort studies (to provide more reliable evidence, we only included prospective cohort studies, which have more advantages than case-control studies); 7) published in English.

Data extraction and quality evaluation

Two investigators respectively reviewed all abstracts for related studies, and read full texts of eligible literatures. We extracted data using a standardized form and assessed study quality. Disagreements were resolved by discussion and consulting a third

For peer review only - http://bmjopen.bmj.com/site/about/guidelines.xhtml

investigator. The following data were collected from each study: 1) publication information: the first author name, publish year; 2) population's characteristics: country of origin, the average age and pre-pregnancy body mass index (BMI), ethnicity, education status, current gestational week of blood sampling, gestation age of infant at birth and season of blood sample; 3) methods: assay methods of serum/plasma vitamin D levels measured and sample size; 4) latitude and the time of year data collected; 5) *OR* as well as their 95% *CI* for each study. If available, the *ORs* with 95% *CI* were collected from the original article. If original important data were unavailable, the *ORs* with 95% *CI* were calculated by using data from observed articles to construct 2×2 tables of low vitamin D status versus the presence or absence of SGA, Otherwise, we contacted the corresponding author by e-mail to obtain further details. Finally, we assessed the eligible studies based on Newcastle-Ottawa Scale (NOS) system. This scale ranging from 0 to 9 contains 9 items (1 point for each) in 3 parts: selection (4 items), comparability (2 items) and exposure or outcomes (3 items). Scores ranging from 0-3 were deemed to poor quality, scores ranging from 4-6 were deemed to moderate quality and scores surpassing 7 were deemed to high quality.

Statistical Analysis

The data extracted from eligible studies were in the form of effect estimate (*OR* or *RR*) and corresponding 95% *CI*. Due to the low level of the morbidity of SGA, the value of *OR* was approximately equal to *RR*.[19] Meta-analysis was performed using STATA package version 12.0 (Stata Corporation, College Station, TX, USA). The *ORs* and 95% *CIs* for normal vitamin D levels versus deficient vitamin D levels from each study were combined to calculate the estimated pooled *OR*, *95% CI* and *P* value. *Q*-statistic test and the I-square (I^2) test were used to estimate the heterogeneity among different studies.[20] The random-effects model was usually a more plausible when studies were gathered from the published literature.[21] Therefore, the random effects model was used for this meta-analysis. To explore the sources of heterogeneity and the various

For peer review only - http://bmjopen.bmj.com/site/about/guidelines.xhtml

results of pre-specified subgroups, subgroup analysis was carried out based on status of cut-off values, study quality (NOS scores), adjustment of critical confounders, sample size, measurement of vitamin D and current gestation of blood sampling. A sensitivity analysis was conducted to determine the stability and reliability of the results by leave one out at a time and checking the consistency of the overall effect estimate. Funnel plots were used to qualitatively assess the publication bias, and Egger's and Begg's tests were also used to quantitatively assess publication bias.[22 23]

RESULTS

Description of included studies

A total of 1734 literatures were identified for initial review using search strategies as described. After removing duplicates, 1 536 studies remained. We screened the titles and abstracts of these studies, excluded 1 518 records according to the inclusion and exclusion. Then the remaining 18 full-text articles were assessed for eligibility. Finally, 13 cohort studies[4 15-18 24-31] were included in the meta-analysis (Figure 1), including 28285 pregnant women.

Characteristics and methodological quality of 13 studies are presented in Table 1 and supplementary Table S1: These studies were published from 2010 to 2016, four of the studies were conducted in USA, three in Netherlands, two in China and one each in Korea, Singapore, Ireland and New Zealand. Of the 13 studies, the average age of the pregnant women was < 30 years old (4 studies) and >30 years old (5 studies), the average pre-pregnancy BMI of 7 studies were below 25 kg/m^2 and 3 studies were above 25 kg/m^2. Nevertheless, 10 studies have adjusted for confounders and 3 studies have not. 5 studies adopted blood during first trimester, 5 studies were second trimester, 3 studies were mixed with first, second or third trimester. Furthermore, 5 different assay methods were used to measure vitamin D levels of pregnant women and 2 different criteria were used for diagnosis of SGA (with the birthweight in the lowest 10th percentile or 15th percentile of the reference population). In addition, the prevalence of maternal vitamin D deficiency varied from 13.2% to 77.3% (showed in

For peer review only - http://bmjopen.bmj.com/site/about/guidelines.xhtml

supplementary Table S1). Finally, the degrees of NOS score were presented as high levels (9 studies) and low levels (4 studies).

Meta-analysis results

The overall results showed that maternal vitamin D deficiency during pregnancy was significantly associated with an increased risk of SGA (pooled *OR*=1.588; *95% CI* 1.138 to 2.216; *P*<0.01) in the random-effects model and the forest plot showed the details (Figure 2).

Subgroup analysis

Due to the existence of heterogeneity (I^2=84.2%; *P*<0.001), subgroup analysis was carried out to explore the possible sources of heterogeneity in the meta-analysis (Table 2). The subgroups were based on status of cut-off values of vitamin D levels, measurement of vitamin D, sample size, study quality (NOS score levels), whether adjusted for critical confounders and gestational week of blood sampling. In subgroup analyses, the confidence intervals were overlapped for each subgroup, which showed no statistically significant difference in the effect estimates. Thus, there were no differences in the association between vitamin D deficiency with SGA based on study quality, gestation of blood sampling, cut-off values, sample size, adjust for critical confounders and measurement of vitamin D (Table 2). However, we did not conduct subgroup analyses of ethnicity, pre-pregnancy BMI, gestational age of infant at birth and season of blood sample due to insufficient/ unspecific data in some studies.

Sensitivity analysis and publication bias

To evaluate the stability of our results, sensitivity analysis was carried out. Chen's study was responsible for most of the heterogeneity in this meta-analysis. Low heterogeneity was observed among the remaining studies (I^2=55.4%, *P*=0.010) and pooled *OR* was 1.336 (95% *CI* 1.103 to 1.692) after excluding Chen's study[4]. Furthermore, there were no obvious changes in the pooled *ORs* as a result of the

For peer review only - http://bmjopen.bmj.com/site/about/guidelines.xhtml

exclusion of any other single study. The pooled *ORs* ranged from 1.366 (95% *CI* 1.103 to 1.692) to 1.693 (95% *CI* 1.211 to 2.366), and each was statistically significant (Table 3). There was also no publication bias after carrying out Begg's test (*P*=0.669) and Egger's regression test (*P*=0.815), the funnel plot showed the details (Figure 3).

DISCUSSION

The prevalence of vitamin D deficiency during pregnancy and its association with risk of SGA caught increasing attentions. Current meta-analysis of prospective cohort studies suggested that vitamin D deficiency is significantly associated with an increased risk of SGA. No publication bias was detected and sensitive analysis showed no single study dramatically influences the results, which indicated that the results of our meta-analysis were stable and reliable.

Our study was in line with several previous studies. A previous meta-analysis showed that low maternal vitamin D levels during pregnancy may be associated with an increased risk of SGA, gestational diabetes mellitus, preterm birth.[5] Similarly, another vital meta-analysis also suggested that vitamin D insufficiency was associated with an increased risk of SGA, preeclampsia, and bacterial vaginosis.[6] However, those studies included both case-control and prospective cohort studies and did not include latest published cohort studies, and did not evaluate the association in specific subgroup analysis. Moreover, the cut-off values for the vitamin D status differed between different studies. Thus, we conducted this meta-analysis to provide stronger evidence for the association between vitamin D and SGA.

Heterogeneity test (the Coherence *Q* test) showed significant heterogeneity existed among studies. We explored the potential influential factors for the results by performing a subgroup analysis. Although the results of subgroup analyses showed that there were no significant differences in the association between vitamin D deficiency with SGA based on study quality, gestation of blood sampling, cut-off values, sample size, adjust for critical confounders and measurement of vitamin D,

For peer review only - http://bmjopen.bmj.com/site/about/guidelines.xhtml

there may be other potential factors contributing to the heterogeneity in our meta-analysis. The different for ethnicity of the maternal, season of blood sample, sunlight exposure and diet during pregnancy are confounding factors for the association between vitamin D deficiency and SGA. Sensitivity analysis showed that exclusion of any single study did not materially alter the overall combined effect, however, our sensitivity suggested that Chen's study probably contributed to the heterogeneity. Therefore, we should look at the results of this meta-analysis objectively.

The underlying mechanism of vitamin D deficiency increases risk of SGA is not entirely clear but might be explained by the inflammatory response. Vitamin D deficiency can increase the levels of proinflammatory cytokines, leading to oxidative stress. Lower 25(OH)D status is associated with increased vascular endothelial cell expression of nuclear factor κB (NFκB) and interleukin 6 (IL-6), and with decreased of vitamin D receptor (VDR) and 1-α hydroxylase.[32] A study reported that the levels of pro-inflammatory cytokines in cord blood of SGA were significantly higher than that in normal-born infants.[33] Mullins et al.[34] showed that the offspring of pregnant women of SGA contained higher tumor necrosis factor (TNF-α) than normal offspring of pregnant women, and as an important inflammatory factor, TNF-α inhibited placental hormone synthesis and stimulated calcitriol catabolism by regulating enzymes.[35] Vitamin D may also play an important role in innate and adaptive immunity by inhibiting the pathway of decidual NFκB to reduce the inflammatory response, since NFκB is a main transcription factor of inflammatory mediators.[36]

Maternal vitamin D deficiency is prevalent, the extent to which can be influenced by many variables including ethnicity, region, skin pigmentation, sun exposure, season, age, vitamin D supplementation and others.[37] The American Association of Endocrinology recommended that pregnant women require at least 600 *IU/d* of vitamin D and confirmed that at least 1500-2000 *IU/d* of vitamin D may be needed to keep a blood level of vitamin D above 30 ng/ml.[38] However, the recommendations of pregnant women vitamin D supplementation is scanty. At present, vitamin D

For peer review only - http://bmjopen.bmj.com/site/about/guidelines.xhtml

supplementation during pregnancy has been suggested as an intervention to prevent adverse pregnancy outcomes.[39] A randomized controlled trial reported that maternal vitamin D supplementation of 2000 and 4000 *IU/d* appeared safe during pregnancy, and the most effective in optimizing serum vitamin D concentrations in mothers and their infants was 4000 *IU/d*,[40] this result was consistent with another randomized controlled trial in Pakistan.[41] Low vitamin D levels during pregnancy could increase the risk of SGA, however, vitamin D supplementation did not significantly reduce the risk of SGA [(*OR*=0.78, *95% CI* 0.50 to 1.21)[42] or (*OR*=0.67,95% *CI* 0.40 to 1.11)[43]]. In addition, it was hard to make final conclusions on need for supplementation of vitamin D during pregnancy.[44] Therefore, we need larger randomized controlled trials to assess the value of these interventions in the future, which has a significant impact on the guidance of the perinatal period care.

Our study has several strengthens. Firstly, to provide more reliable evidence, we only included prospective cohort studies, which have more advantages than case-control studies as we all familiar with. Secondly, no publication bias existing, indicating that the included results may be unbiased and credible. At last, subgroups analysis of our study presented more thorough understanding of current evidence. However, several limitations should also be acknowledged. The association between maternal vitamin D status and SGA risk could be affected by confounding factors such as pre-pregnancy BMI, age, education, race and exposure sunlight, however, not all studies are control these confounding factors in our meta-analysis. Then, different definition of vitamin D deficiency, insufficiency or sufficiency might have influenced the result. Lastly, pooled data without detail individual information were used to performed meta-analysis, which restricted us to get more comprehensive results.

CONCLUSIONS

The present study indicates that low vitamin D levels is associated with an increased risk of SGA. Further confirmation of these findings in larger sample size studies are required. The role of vitamin D in the pathogenesis of SGA should be emphasized. As

For peer review only - http://bmjopen.bmj.com/site/about/guidelines.xhtml

well, early screening for vitamin D deficiency among pregnant women may be necessary.

Contributors FT contributed to study design; SL, XW and BZ contributed analysis tools and methods; YC analyzed the data and drafted the manuscript; BZ and FT revised the manuscript. All authors read and approved the final version of the manuscript.

Funding This work is grateful for the financial support offered by the National Natural Science Foundation of China (81330068, 81573168)

Data sharing statement No additional data are available

Competing interests None declared.

For peer review only

For peer review only - http://bmjopen.bmj.com/site/about/guidelines.xhtml

REFERENCES

1. Finberg L. Vitamin D deficiency and rickets. *J Pediatr Endocrinol Metab* 2006; 19:203.
2. Palacios C, Gonzalez L. Is vitamin D deficiency a major global public health problem? *J Steroid Biochem Mol Biol* 2014; 144 Pt A:138-145.
3. Eggemoen AR, Falk RS, Knutsen KV, *et al*. Vitamin D deficiency and supplementation in pregnancy in a multiethnic population-based cohort. *BMC pregnancy and childbirth* 2016; 16:7.
4. Chen YH, Fu L, Hao JH, *et al*. Maternal vitamin D deficiency during pregnancy elevates the risks of small for gestational age and low birth weight infants in Chinese population. *J Clin Endocrinol Metab* 2015; 100:1912-1919.
5. Wei SQ, Qi HP, Luo ZC, *et al*. Maternal vitamin D status and adverse pregnancy outcomes: a systematic review and meta-analysis. *J Matern Fetal Neonatal Med* 2013; 26:889-899.
6. Aghajafari F, Nagulesapillai T, Ronksley PE, *et al*. Association between maternal serum 25-hydroxyvitamin D level and pregnancy and neonatal outcomes: systematic review and meta-analysis of observational studies. *BMJ* 2013; 346:f1169.
7. Mikolajczyk RT, Zhang J, Betran AP, *et al*. A global reference for fetal-weight and birthweight percentiles. *Lancet* 2011; 377:1855-1861.
8. Hoftiezer L, Hukkelhoven CW, Hogeveen M, *et al*. Defining small-for-gestational-age: prescriptive versus descriptive birthweight standards. *Eur J Pediatr* 2016; 175:1047-1057.
9. Ding G, Tian Y, Zhang Y, *et al*. Application of a global reference for fetal-weight and birthweight percentiles in predicting infant mortality. *BJOG* 2013; 120(13):1613-1621.
10. Christian P, Murray-Kolb LE, Tielsch JM, *et al*. Associations between preterm birth, small-for-gestational age, and neonatal morbidity and cognitive function among school-age children in Nepal. *BMC pediatrics* 2014; 14:58.
11. Katz J, Lee AC, Kozuki N, *et al*. Mortality risk in preterm and small-for-gestational-age infants in low-income and middle-income countries: a pooled country analysis. *Lancet* 2013; 382(9890):417-25.
12. de Zegher F, Sebastiani G, Diaz M, *et al*. Body composition and circulating high-molecular-weight adiponectin and IGF-I in infants born small for gestational age: breast- versus formula-feeding. *Diabetes* 2012; 61:1969-1973.
13. Wolfenstetter A, Simonetti GD, Poschl J, *et al*. Altered cardiovascular rhythmicity in children born small for gestational age. *Hypertension* 2012; 60:865-870.
14. Aly H, Davies J, El-Dib M, *et al*. Renal function is impaired in small for gestational age premature infants. *J Matern Fetal Neonatal Med* 2013; 26:388-391.
15. Leffelaar ER, Vrijkotte TG, van Eijsden M. Maternal early pregnancy vitamin D status in relation to fetal and neonatal growth: results of the multi-ethnic Amsterdam Born Children and their Development cohort. *Br J Nutr* 2010; 104:108-117.
16. Gernand AD, Simhan HN, Klebanoff MA, *et al*. Maternal serum 25-hydroxyvitamin D and measures of newborn and placental weight in a U.S. multicenter cohort study. *J Clin Endocrinol Metab* 2013; 98:398-404.
17. Nobles CJ, Markenson G, Chasan-Taber L. Early pregnancy vitamin D status and risk for adverse maternal and infant outcomes in a bi-ethnic cohort: the Behaviors Affecting Baby and You (B.A.B.Y.) Study. *Br J Nutr* 2015; 114:2116-2128.

For peer review only - http://bmjopen.bmj.com/site/about/guidelines.xhtml

18. Ong YL, Quah PL, Tint MT, *et al*. The association of maternal vitamin D status with infant birth outcomes, postnatal growth and adiposity in the first 2 years of life in a multi-ethnic Asian population: the Growing Up in Singapore Towards healthy Outcomes (GUSTO) cohort study. *Br J Nutr* 2016, 116:621-631.

19. Gagne JJ, Power MC. Anti-inflammatory drugs and risk of Parkinson disease: a meta-analysis. *Neurology* 2010; 74:995-1002.

20. Takkouche B, Cadarso-Suarez C, Spiegelman D. Evaluation of old and new tests of heterogeneity in epidemiologic meta-analysis. *Am J Epidemiol* 1999; 150:206-215.

21. Borenstein M, Hedges LV, Higgins JP, *et al*. A basic introduction to fixed-effect and random-effects models for meta-analysis. *Res Synth Methods* 2010; 1(2):97-111.22. Egger M, Davey Smith G, Schneider M, *et al*. Bias in meta-analysis detected by a simple, graphical test. *BMJ* 1997; 315:629-634.

23. Begg CB, Mazumdar M. Operating characteristics of a rank correlation test for publication bias. *Biometrics* 1994; 50:1088-1101.

24. Burris HH, Rifas-Shiman SL, Camargo CA, *et al*. Plasma 25-hydroxyvitamin D during pregnancy and small-for-gestational age in black and white infants. *Ann Epidemiol* 2012; 22:581-586.

25. Zhou J, Su L, Liu M, *et al*. Associations between 25-hydroxyvitamin D levels and pregnancy outcomes: a prospective observational study in southern China. *Eur J Clin Nutr* 2014; 68:925-930.

26. Choi R, Kim S, Yoo H, *et al*. High prevalence of vitamin D deficiency in pregnant Korean women: the first trimester and the winter season as risk factors for vitamin D deficiency. *Nutrients* 2015; 7:3427-3448.

27. Kiely ME, Zhang JY, Kinsella M, *et al*. Vitamin D status is associated with uteroplacental dysfunction indicated by pre-eclampsia and small-for-gestational-age birth in a large prospective pregnancy cohort in Ireland with low vitamin D status. *Am J Clin Nutr* 2016; 104:354-361.

28. Scholl TO, Chen X, Stein TP. Maternal calcium metabolic stress and fetal growth. *Am J Clin Nutr* 2014; 99:918-925.

29. Boyle VT, Thorstensen EB, Mourath D, *et al*. The relationship between 25-hydroxyvitamin D concentration in early pregnancy and pregnancy outcomes in a large, prospective cohort. *Br J Nutr* 2016; 116:1409-1415.

30. van den Berg G, van Eijsden M, Vrijkotte TG, *et al*. Suboptimal maternal vitamin D status and low education level as determinants of small-for-gestational-age birth weight. *Eur J Nutr* 2013; 52:273-279.

31. Miliku K, Vinkhuyzen A, Blanken LM, *et al*. Maternal vitamin D concentrations during pregnancy, fetal growth patterns, and risks of adverse birth outcomes. *Am J Clin Nutr* 2016; 103:1514-1522.

32. Jablonski KL, Chonchol M, Pierce GL, *et al*. 25-Hydroxyvitamin D deficiency is associated with inflammation-linked vascular endothelial dysfunction in middle-aged and older adults. *Hypertension* 2011; 57:63-69.

33. Lausten-Thomsen U, Olsen M, Greisen G, *et al*. Inflammatory markers in umbilical cord blood from small-for-gestational-age newborns. *Fetal Pediatr Pathol* 2014; 33:114-118.

34. Mullins E, Prior T, Roberts I, *et al*. Changes in the maternal cytokine profile in pregnancies complicated by fetal growth restriction. *Am J Reprod Immunol* 2012; 68:1-7.

For peer review only - http://bmjopen.bmj.com/site/about/guidelines.xhtml

35. Diaz L, Noyola-Martinez N, Barrera D, *et al*. Calcitriol inhibits TNF-alpha-induced inflammatory cytokines in human trophoblasts. *J Reprod Immunol* 2009; 81:17-24.

36. Cohen-Lahav M, Shany S, Tobvin D, *et al*. Vitamin D decreases NFkappaB activity by increasing IkappaBalpha levels. *Nephrol Dial Transplant* 2006; 21:889-897.

37. Bodnar LM, Simhan HN. Vitamin D may be a link to black-white disparities in adverse birth outcomes. *Obstet Gynecol Surv* 2010; 65:273-284.

38. Holick MF, Binkley NC, Bischoff-Ferrari HA, *et al*. Evaluation, treatment, and prevention of vitamin D deficiency: an Endocrine Society clinical practice guideline. *J Clin Endocrinol Metab* 2011; 96:1911-1930.

39. De-Regil LM, Palacios C, Lombardo LK, *et al*. Vitamin D supplementation for women during pregnancy. *Sao Paulo Med* 2016; 134:274-275.

40. Dawodu A, Saadi HF, Bekdache G, *et al*. Randomized controlled trial (RCT) of vitamin D supplementation in pregnancy in a population with endemic vitamin D deficiency. *J Clin Endocrinol Metab* 2013; 98:2337-2346.

41. Hossain N, Kanani FH, Ramzan S, *et al*. Obstetric and neonatal outcomes of maternal vitamin D supplementation: results of an open-label, randomized controlled trial of antenatal vitamin D supplementation in Pakistani women. *J Clin Endocrinol Metab* 2014; 99:2448-2455.

42. Perez-Lopez FR, Pasupuleti V, Mezones-Holguin E, *et al*. Effect of vitamin D supplementation during pregnancy on maternal and neonatal outcomes: a systematic review and meta-analysis of randomized controlled trials. *Fertil Steril* 2015; 103:1278-1288 e1274.

43. Thorne-Lyman A, Fawzi WW. Vitamin D during pregnancy and maternal, neonatal and infant health outcomes: a systematic review and meta-analysis. *Paediatr Perinat Epidemiol* 2012; 26:75-90

44. Karras SN, Fakhoury H, Muscogiuri G, *et al*. Maternal vitamin D levels during pregnancy and neonatal health: evidence to date and clinical implications. *Ther Adv Musculoskelet Dis* 2016; 8(4):124-35.

For peer review only

For peer review only - http://bmjopen.bmj.com/site/about/guidelines.xhtml

Table 1. Characteristics of the included studies in the present meta-analysis

Author	Region	Year	Age at baseline (mean, year)	Pre-pregnancy BMI (mean, kg/m^2)	Gestational week of blood sampling	Measurement of vitamin D	SGA criteria	Cut-off values	Ethnicity group	OR (95% CI)	Adjusted	NOS Score	Sample size
Leffelaar[15]	Netherlands	2010	NA	NA	12-14 weeks	enzyme immunoassay	$<10^{th}$	< 15 ng/ml	Dutch (60.3%), Surinamese (6.7%), Turkish (4.0%), Moroccan (6.3%), Other non-western (14.2%), Other western (8.6%)	1.90(1.40,2.70)	yes	8	3730
Burris[24]	USA	2012	32.5	24.8	26-28 weeks	CLIA and RIA	$<10^{th}$	< 10 ng/ml	White (83.6%), Black (16.4%)	3.17(1.16,8.63)	yes	7	1133
Zhou[25]	China	2014	29.5	20.3	16-20 weeks	ECLIA	$<10^{th}$	< 20 ng/ml	Asian	2.46(0.71,8.46)	no	8	1923
Choi[26]	Korea	2015	32.0	20.2	first or second or third trimester	LC-MS/MS	$<10^{th}$	< 20 ng/ml	Asian	0.448(0.149,1.351)	yes	6	220
Ong[18]	Singapore	2016	30.5	26.1	26-28 weeks	LC-MS/MS	$<10^{th}$	< 20 ng/ml	Asian	1.00(0.56,1.79)	yes	8	910
Kiely[27]	Ireland	2016	30.5	24.9	14-16 weeks	LC-MS/MS	$<10^{th}$	< 20 ng/ml	White (98%), Others (2%)	0.88(0.60,1.28)	yes	6	1768
Scholl[28]	USA	2014	22.8	26	13.8±5.6 weeks	HPLC	$<10^{th}$	< 20 ng/ml	Hispanic (51.4%), Non-Hispanic black (34.4%), Non-Hispanic white (14.2%)	0.930(0.568,1.523)	no	8	1045
Chen[4]	China	2015	27.5	NA	first or second or third trimester	RIA	$<10^{th}$	< 20 ng/ml	Asian	6.47(4.30,9.75)	yes	6	3658
Boyle[29]	New Zealand	2016	30.3	24.8	15 weeks	LC-MS/MS	$<10^{th}$	< 20 ng/ml	NZ European (83.8%), other ethnicities (16.2%)	1.33(0.91,1.96)	yes	7	2065
Berg[30]	Netherlands	2013	NA	NA	12.9 weeks	enzyme immunoassay	$<10^{th}$	< 20 ng/ml	NA	1.57(1.03,2.39)	yes	7	2274
Gerand[16]	USA	2013	NA	22.3	20.6 weeks	LC-MS/MS	$<10^{th}$	<15 ng/ml	White (52.1%), Black (41.6%), Puerto Rican (6.3%)	1.284(1.026,1.608)	no	6	2146
Miliku[31]	Netherlands	2016	29.7	23.7	20.3 weeks	LC-MS/MS	$<15^{th}$	<10 ng/ml	European (57.3%), Cape Verdean (4.4%), Dutch Antillean (3.5%), Moroccan (6.6%), Surinamese (9.1%), Turkish (9.2%), Other (9.9%)	2.07(1.33,3.22)	yes	7	7176
Nobles[17]	USA	2015	NA	>25	first or second or third trimester	ECLIA	$<10^{th}$	< 20 ng/ml	[White (75.6%), Black (13.5%)]	2.14(0.67,6.88)	yes	8	237

For peer review only

CLIA: chemiluminescence immunoassay; RIA: radioimmunoassay; ECLIA: electrochemiluminescence immunoassay; LC-MS/MS: liquid chromatography-tandem mass spectrometry; NA: not available.

For peer review only

For peer review only - http://bmjopen.bmj.com/site/about/guidelines.xhtml

Table 2. Subgroup analysis of the association between maternal Vitamin D deficiency and SGA

Stratification group	N	P Value for OR	OR (95% CI)	Heterogeneity test	
				I-square (%)	P Value
Study quality (NOS)					
High	9[15 17 18 24 25 28-31]	<0.001	1.555 (1.239, 1.951)	37.6	0.118
Low	4[4 16 26 27]	0.440	1.441 (0.570, 3.641)	95.2	<0.001
Gestation of blood sampling					
first trimester	5[15 27 28 29 30]	0.104	1.286 (0.950,1.741)	65.9	0.020
second trimester	5[16 18 24 25 31]	0.011	1.577 (1.110, 2.240)	51.1	0.085
Mixed (first or second or third)	3[4 17 26]	0.432		90.6	<0.001
Cut-off values					
＜10 ng/ml	2[24 31]	0.001	2.219 (1.480, 3.325)	0	0.446
＜15 ng/ml	2[15 16]	0.029	1.532(1.046, 2.246)	73.2	0.054
＜20 ng/ml	9[4 17 18 25-30]	0.172	1.448 (0.851, 2.465)	88.2	＜0.001
Sample size					
> 1000	10[4 15 16 24 25 27-31]	0.003	1.760(1.217, 2.544)	86.8	<0.001
< 1000	3[17 18 26]	0.946	0.975(0.476, 1.999)	45.5	0.160
Adjust for critical confounders					
yes	10[4 15 17 18 24 26 29-31]	0.018	1.681 (1.094, 2.584)	86.3	< 0.001
no	3[16 25 28]	0.180	1.219 (0.912, 1.629)	22.3	0.276
Measurement of vitamin D					
LC-MS/MS	6[16 18 26 27 29 31]	0.204	1.195 (0.908, 1.573)	59.5	0.031
Others	7[4 15 17 24 25 28 29]	0.006	2.224 (1.263, 3.918)	85.8	<0.001

For peer review only

For peer review only - http://bmjopen.bmj.com/site/about/guidelines.xhtml

For peer review only

For peer review only - http://bmjopen.bmj.com/site/about/guidelines.xhtml

Table 3. Sensitivity analyses of the association between vitamin D deficiency and SGA

Study omitted	*OR* (95% CI)	*P* value	I-square (%)	*P* value
Leffelaar[15]	1.559 (1.074, 2.263)	0.020	85.2	< 0.001
Burris[24]	1.527 (1.08, 2.152)	0.016	85.1	< 0.001
Zhou[25]	1.557 (1.105, 2.195)	0.011	85.4	< 0.001
Choi[26]	1.693 (1.211, 2.366)	0.002	84.5	< 0.001
Ong[18]	1.652 (1.162, 2.350)	0.005	85.0	< 0.001
Kiely[27]	1.686 (1.191, 2.387)	0.003	83.4	< 0.001
Scholl[28]	1.669 (1.174, 2.371)	0.004	84.6	< 0.001
Chen[4]	1.366 (1.103, 1.692)	0.004	55.4	0.010
Boyle[29]	1.616 (1.118, 2.335)	0.011	85.4	< 0.001
Berg[30]	1.590 (1.102, 2.293)	0.013	85.4	< 0.001
Gerand[16]	1.624 (1.100, 2.397)	0.015	84.7	< 0.001
Miliku[31]	1.548 (1.079, 2.220)	0.018	85.1	< 0.001

For peer review only - http://bmjopen.bmj.com/site/about/guidelines.xhtml

Nobles[17]	1.565 (1.109, 2.209)	0.011	85.4	< 0.001

For peer review only

For peer review only - http://bmjopen.bmj.com/site/about/guidelines.xhtml

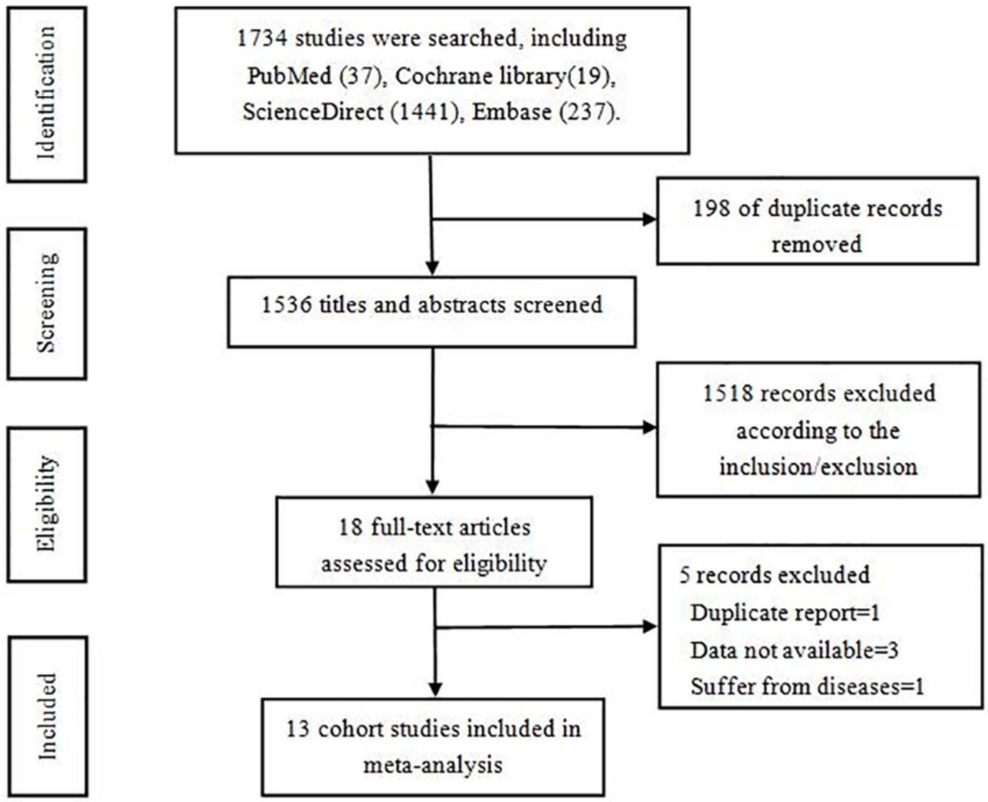

Figure1. Flowchart of the literature search and trial selection process

91x74mm (300 x 300 DPI)

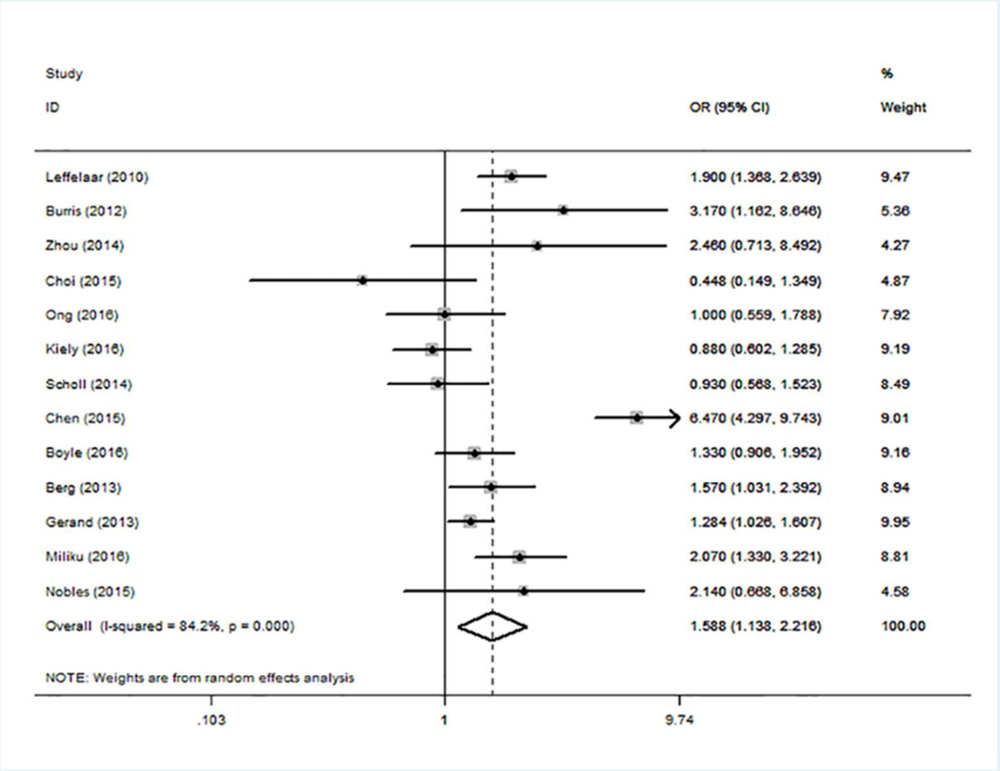

Figure 2. Forest plots of summary crude odds ratios of the association between vitamin D deficiency

91x70mm (300 x 300 DPI)

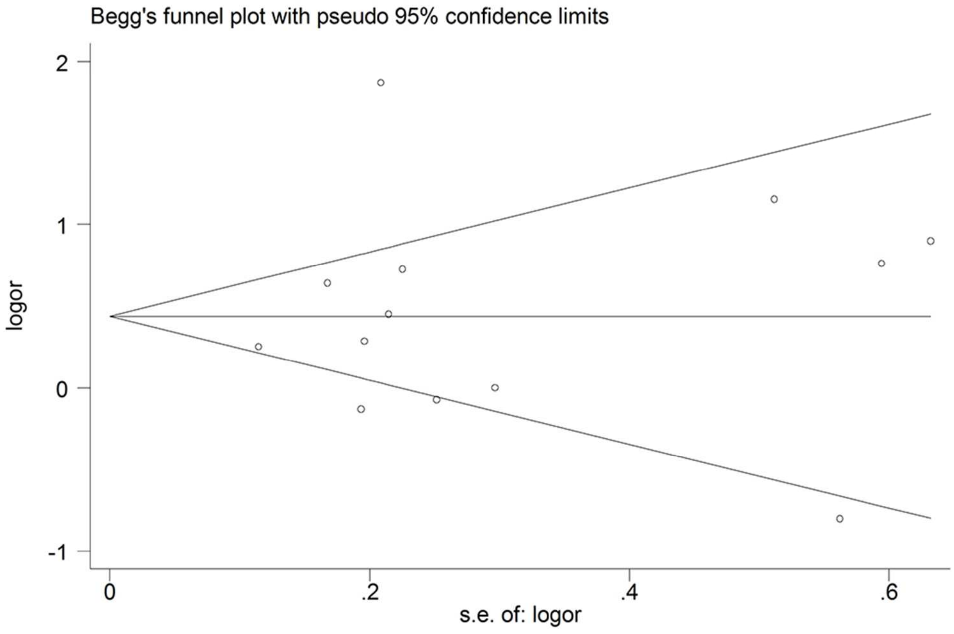

Figure 3. Funnel plot for small for gestational age. Log OR of the individual studies plotted against the standard error of log OR.

91x60mm (300 x 300 DPI)

For peer review only

Supplementary Box S1. The search strategy of PubMed

#1 (vitamin D) OR (25-hydroxyvitamin D) OR cholecalciferol OR (25(OH)D)

#2 (small for gestational age) OR (small-for-gestation age) OR (small size for gestational age) OR SGA

#3 #1 AND #2

For peer review only

Supplementary Table S1. Characteristics of the included studies in the present meta-analysis

Author	Latitude	The time of year data collected	Gestational age of infant at birth	The prevalence of SGA	The prevalence of vitamin D deficiency	Maternal education status	Season of blood sample
Leffelaar[15]	NA	2003.2~2004.3	40.1±1.2 weeks	9.2%	23.1%	≤5 years (17.2%), 6-10years (38.5%), ≥11 years (44.3%)	Summer (43.6%)
Burris[24]	NA	NA	39.6 weeks	4.8%	32.4%	College graduate (41.2%)	NA
Zhou[25]	23.1°N	2010.9~2011.8	NA	0.6%	18.9%	NA	NA
Choi[26]	36.0°N	2012.4~2013.9	NA	10.9%	77.3%	≤12 years(5.5%), > 12 years(94.5%)	Spring (44.5%), Summer (10.0%), Fall (39.5%), Winter (5.9%)
Ong[18]	1°22′N	NA	NA	9.1%	13.2%	Primary and secondary (30.2%), Post-secondary (35.4%), University (34.4%)	NA
Kiely[27]	52°N	2008.3~2011.2	NA	10.7%	44%	Secondary (61%), Tertiary (39%)	Winter (58.5%), Summer (41.5%)
Scholl[28]	NA	2001~2007	38.5	7.2%	33.7%	NA	NA
Chen[4]	31°52′N	2008.11~2010.10	NA	8.9%	38.41%	NA	Spring (36.7%), Summer (22.5%), Fall (20.6%), Winter (20.2%)
Boyle[29]	NA	2005~2008	NA	9.9%	21.5%	NA	Spring (20.5%), Summer (26.4%), Fall (23.2%), Winter (29.8%)
Berg[30]	52°22′N	2003.2~2004.3	20-42 weeks	9.1%	NA	NA	Winter (55.5%)
Gerand[16]	≥41°N(63.0%), 38~40°N(28.8%), ≤35°N(8.2%)	1959~1965	39.7±1.3 weeks	18.4%	34.8%	NA	Spring (25.9%), Summer (25.7%), Fall (24.6%), Winter (23.9%)
Miliku[31]	NA	2002.4~2006.1	35.9-42.3 weeks	5.0%	53.2%	No higher education (59.2%), Higher education (40.8%)	Spring (29.5%), Summer (22.9%), Fall (24.0%), Winter (23.6%)
Nobles[17]	NA	2007~2012	NA	9.6%	20.7%	≤High school (55.2%), > High school (44.8%)	Summer (41.4%), Winter (58.7%)

NA：not available.

For peer review only

Supplementary Box S2. Quality assessment of cohort studies

NEWCASTLE - OTTAWA QUALITY ASSESSMENT SCALE

COHORT STUDIES

Note: A study can be awarded a maximum of one star for each numbered item within the Selection and Outcome categories. A maximum of two stars can be given for Comparability

Selection

1) Representativeness of the exposed cohort

a) truly representative of the average _______________ (describe) in the community ✵

b) somewhat representative of the average ______________ in the community ✵

c) selected group of users eg nurses, volunteers

d) no description of the derivation of the cohort

2) Selection of the non-exposed cohort

a) drawn from the same community as the exposed cohort ✵

b) drawn from a different source

c) no description of the derivation of the non-exposed cohort

3) Ascertainment of exposure

a) secure record (eg surgical records) ✵

b) structured interview ✵

c) written self-report

d) no description

4) Demonstration that outcome of interest was not present at start of study

a) yes ✵

b) no

Comparability

1) Comparability of cohorts on the basis of the design or analysis

a) study controls for _____________ (select the most important factor) ✵

b) study controls for any additional factor ✵ (This criteria could be modified to indicate specific control for a second important factor.)

Outcome

1) Assessment of outcome

a) independent blind assessment ✵

b) record linkage ✵

c) self-report

d) no description

2) Was follow-up long enough for outcomes to occur

a) yes (select an adequate follow up period for outcome of interest) ✵

b) no

3) Adequacy of follow up of cohorts

a) complete follow up - all subjects accounted for ✵

b) subjects lost to follow up unlikely to introduce bias - small number lost - > ____ % (select an adequate %) follow up, or description provided of those lost) ✵

c) follow up rate < ____% (select an adequate %) and no description of those lost

d) no statement

For peer review only

Supplementary Table S2. Quality scores of included studies on vitamin D status and SGA.

Study	Selection				Comparability	Outcome			Total scores
	Representativeness of the exposed cohort	Selection of the non-exposed cohort	Ascertainment of exposure	Demonstration that outcome of interest was not present at start of study	Comparability of cohorts on the basis of design or analysis	Assessment of outcome	Was follow-up long enough for outcomes to occur	Adequacy of follow up of cohorts	
Leffelaar[14]	1	1	1	1	1	1	1	1	8
Burris[23]	1	1	1	1	1	1	1		7
Zhou[24]	1	1	1	1	1	1	1	1	8
Choi[25]	1	1	1	1	1	1			6
Ong[17]	1	1	1	1	2	1	1		8
Kiely[26]	1		1	1	1	1	1		6
Scholl[27]	1	1	1	1	2		1	1	8
Chen[4]	1	1	1	1		1	1		6
Boyle[28]	1	1	1	1	1	1	1		7
Berg[29]	1	1	1	1	1	1	1		7
Gerand[15]	1	1	1	1		1	1		6
Miliku[30]	1	1	1	1	1	1	1		7
Nobles[16]	1	1	1	1	1	1	1	1	8

For peer review only

For peer review only

Section and topic	Item No	Checklist item	Reported on page No
Title:			
Identification	1a	Identify the report as a protocol of a systematic review	1
Update	1b	If the protocol is for an update of a previous systematic review, identify as such	
Registration	2	If registered, provide the name of the registry (such as PROSPERO) and registration number	
Authors:			
Contact	3a	Provide name, institutional affiliation, e-mail address of all protocol authors; provide physical mailing address of corresponding author	1
Contributions	3b	Describe contributions of protocol authors and identify the guarantor of the review	10
Amendments	4	If the protocol represents an amendment of a previously completed or published protocol, identify as such and list changes; otherwise, state plan for documenting important protocol amendments	
Support:			
Sources	5a	Indicate sources of financial or other support for the review	10
Sponsor	5b	Provide name for the review funder and/or sponsor	
Role of sponsor or funder	5c	Describe roles of funder(s), sponsor(s), and/or institution(s), if any, in developing the protocol	
**INTRODUCTION**			
Rationale	6	Describe the rationale for the review in the context of what is already known	3
Objectives	7	Provide an explicit statement of the question(s) the review will address with reference to participants, interventions, comparators, and outcomes (PICO)	3
**METHODS**			
Eligibility criteria	8	Specify the study characteristics (such as PICO, study design, setting, time frame) and report characteristics (such as years considered, language, publication status) to be used as criteria for eligibility for the review	4
Information sources	9	Describe all intended information sources (such as electronic databases, contact with study authors, trial registers or other grey literature sources) with planned dates of coverage	3
Search strategy	10	Present draft of search strategy to be used for at least one electronic database, including planned limits, such that it could be repeated	3

For peer review only

Study records:			
Data management	11a	Describe the mechanism(s) that will be used to manage records and data throughout the review	8
Selection process	11b	State the process that will be used for selecting studies (such as two independent reviewers) through each phase of the review (that is, screening, eligibility and inclusion in meta-analysis)	4,18
Data collection process	11c	Describe planned method of extracting data from reports (such as piloting forms, done independently, in duplicate), any processes for obtaining and confirming data from investigators	4,18
Data items	12	List and define all variables for which data will be sought (such as PICO items, funding sources), any pre-planned data assumptions and simplifications	14
Outcomes and prioritization	13	List and define all outcomes for which data will be sought, including prioritization of main and additional outcomes, with rationale	14-16
Risk of bias in individual studies	14	Describe anticipated methods for assessing risk of bias of individual studies, including whether this will be done at the outcome or study level, or both; state how this information will be used in data synthesis	16
Data synthesis	15a	Describe criteria under which study data will be quantitatively synthesised	5
	15b	If data are appropriate for quantitative synthesis, describe planned summary measures, methods of handling data and methods of combining data from studies, including any planned exploration of consistency (such as I^2, Kendall's τ)	5
	15c	Describe any proposed additional analyses (such as sensitivity or subgroup analyses, meta-regression)	15
	15d	If quantitative synthesis is not appropriate, describe the type of summary planned	5
Meta-bias(es)	16	Specify any planned assessment of meta-bias(es) (such as publication bias across studies, selective reporting within studies)	7
Confidence in cumulative evidence	17	Describe how the strength of the body of evidence will be assessed (such as GRADE)	7,9,16,20

For peer review only

BMJ Open

Association between maternal vitamin D deficiency and small for gestational age: evidence from a meta-analysis of prospective cohort studies

Journal:	*BMJ Open*
Manuscript ID	bmjopen-2017-016404.R2
Article Type:	Research
Date Submitted by the Author:	30-Jun-2017
Complete List of Authors:	Chen, Yao; Anhui Medical University, School of Public Health<br>Zhu, Beibei; Anhui Medical University, School of Public Health<br>Wu, Xiaoyan<br>Li, Si; Anhui Medical University, School of Public Health<br>Tao, Fangbiao; Anhui Provincial Key Laboratory of Population Health and Aristogenics,
**Primary Subject Heading**:	Public health
Secondary Subject Heading:	Nutrition and metabolism
Keywords:	vitamin D, small for gestational age, cohort study, meta-analysis

SCHOLARONE™ Manuscripts

For peer review only

Association between maternal vitamin D deficiency and small for gestational age: evidence from a meta-analysis of prospective cohort studies

Yao Chen[1], Beibei Zhu[1,2], Xiaoyan Wu[1,2], Si Li[3], Fangbiao Tao[1,2]

[1]Department of Maternal, Child and Adolescent Health, School of Public Health, Anhui Medical University, Hefei, China

[2]Anhui Provincial Key Laboratory of Population Health and Aristogenics, Hefei, China

[3]Department of Epidemiology and Biostatistics, School of Public Health, Anhui Medical University, Hefei, China.

Correspondence to: Fangbiao Tao, Department of Maternal, Child and Adolescent Health, School of Public Health, Anhui Medical University, 81Meishan Road, Hefei, Anhui, 230032, P.R.China. Tel.: +86 551 65161169.

Email: taofangbiao@126.com

For peer review only

For peer review only - http://bmjopen.bmj.com/site/about/guidelines.xhtml

ABSTRACT

Objective: To determine whether maternal vitamin D deficiency during pregnancy is associated with small for gestational age (SGA).

Methods: A comprehensive literature search of PubMed, the Cochrane Library, Embase, and the Elsevier ScienceDirect library was conducted to identify relevant articles reporting prospective cohort studies in English, with the last report included published in February 2017. Pooled odds ratios (*OR*s) and corresponding 95% confidence intervals (*CI*s) were used to evaluate the correlation in a random effects model.

Results: A total of 13 cohort studies were included in this meta-analysis with a sample of 28285 individuals from seven countries. The pooled overall *OR* for babies with born SGA was 1.588 (*95% CI* 1.138 to 2.216; $P < 0.01$) for women with vitamin D deficiency. The prevalence of vitamin D deficiency during pregnancy varied from 13.2% to 77.3%. Subgroup analyses identified no significant differences in the association between vitamin D deficiency and SGA based on study quality, gestational week during which blood sampling was performed, cut-off vitamin D levels, sample size, adjustment for critical confounders and method for measuring vitamin D.

Conclusion: This meta-analysis suggests that vitamin D deficiency is associated with an increased risk of SGA.

Keywords: vitamin D; small for gestational age; cohort study; meta-analysis.

Strengths and limitations of this study: 1) To our knowledge, this was the first systematic review that included only prospective cohort studies in its evaluation of the association between vitamin D and SGA. 2) The subgroup analysis performed in this study enabled more thorough understanding of current evidence. 3) Cohort study quality tests, a heterogeneity test, and sensitivity analysis were performed; publication bias was evaluated. 4) Different definitions of vitamin D deficiency, insufficiency, or sufficiency may have affected the results. 5) Substantial heterogeneity existed among several outcomes.

For peer review only

INTRODUCTION

Vitamin D is fat-soluble and a steroid hormone recognized for its major role in calcium metabolism and bone health.[1] Vitamin D deficiency or insufficiency has become a global public health issue,[2] especially for pregnant women, among whom the highest deficiency rate is 84% according to a multiethnic population survey conducted in Norway.[3] Several large-population studies have evaluated the associations of maternal vitamin D deficiency with various adverse maternal and fetal outcomes[4-6] including small for gestational age (SGA).

SGA infants are defined as smaller in size than normal for the gestational age, most commonly stipulated by a weight less than the 10th percentile for the corresponding gestational age.[7 8] The incidence of SGA infants worldwide is 9.7%[9], and this percentage is increasing. Infants born SGA have much higher neonatal morbidity and mortality.[10] Katz et al.[11] demonstrated that the pooled risk ratios (*RR*s) of neonatal mortality and post-neonatal morbidity in infants who were SGA were 1.83 and 1.90, respectively. SGA infants may also be strongly correlated with adverse health outcomes in adult life, such as neurocognitive impairment, poor school performance, short stature, and increased risks of diabetes,[12] cardiovascular disease,[13] and kidney disease.[14]

Although numerous studies have focused on the association between maternal vitamin D status and SGA, the results of these studies remain inconsistent. A prospective cohort study conducted in the Netherlands evaluated vitamin D concentrations in 3,730 pregnant women after 12-14 weeks of gestation and discovered that infants born to mothers with vitamin D deficiency had an increased risk of being SGA compared with those born to mothers with adequate vitamin D levels.[15] Subsequently, Gernand et al.[16] reported that if the maternal vitamin D level was less than 15 ng/mL, infants had a significantly higher risk of being SGA. However, other studies have identified no association between vitamin D status and SGA.[17 18]

Given the inconclusive evidence regarding this issue, we summarize the highest

For peer review only - http://bmjopen.bmj.com/site/about/guidelines.xhtml

quality evidence currently available on the basis of a meta-analysis of prospective cohort studies to determine whether vitamin D deficiency in pregnant women is associated with SGA.

MATERIALS AND METHODS

Data sources, search strategy, and selection criteria

A systematic literature search was performed using the PubMed, Elsevier ScienceDirect, Cochrane Library, and Embase databases to identify all relevant articles published prior to March 2017. No restrictions were placed regarding maternal age, and study design. The following keywords were used: ‘vitamin D’ or ‘cholecalciferol’ or ‘25-hydroxyvitamin D’ or ‘25(OH)D’ combined with ‘SGA’ or ‘small for gestational age’ or ‘small-for-gestation-age’ or ‘small size for gestational age’ (see online supplementary box S1 details the search strategy)

Selection criteria

We first screened the titles and abstracts of all the articles to identify possible eligible studies, and then read the articles in full to determine whether they were in fact eligible. The articles included in the meta-analysis were selected according to the following inclusion criteria: (1) published in English; (2) the population of the study was pregnant women without pre-chronic disease; (3) only women with singleton gestation were included; (4) the outcome was SGA infant, the control group included women who gave birth to babies not SGA, and the exposure was ‘vitamin D deficiency’ [25(OH)D < 20ng/mL]; (5) study data were in the form of effect estimates [odds ratio (*OR*) or *RR*] and corresponding 95% confidence intervals (*CI*), or the article reported data that enable calculation of these; (6) maternal blood samples were taken for assessing 25(OH)D during pregnancy; (7) the study design was that of a cohort study. The final criterion was applied because cohort studies are the most effective means of ascertaining both the incidence and natural history of a disorder. The temporal connection between putative cause and outcome is usually clear in such studies; in addition, the cohort study design reduces the risk of survivor bias. By

For peer review only - http://bmjopen.bmj.com/site/about/guidelines.xhtml

contrast, this bias often frustrates cross-sectional and case-control studies. For example, case-control studies are more prone to recall and selection biases and are uncertain regarding chronological order, making them of limited use for causal inference.

Data extraction and quality evaluation

Two investigators reviewed all abstracts of related articles, and read their full text, respectively. We extracted data using a standardized form and assessed study quality. Disagreements were resolved by discussion and consulting a third investigator. The following data were collected from each study: (1) publication information: first author name, and publication year; (2) population's characteristics: country of origin, average age and pre-pregnancy body mass index (BMI), ethnicity, education status, current gestational week of blood sampling, gestational age of infant at birth, and season of blood sample; (3) methods: assay of serum or plasma vitamin D levels and sample size; (4) latitude and time of year that data were collected; (5) *OR* and corresponding 95% *CI* for each study. If available, *OR*s with 95% *CI*s were collected from the original article. If crucial original data were unavailable, *OR*s with 95% *CI*s were calculated using other data published in the article to construct 2 × 2 tables of low vitamin D status versus the presence or absence of SGA, Otherwise, we contacted the corresponding author by e-mail to obtain further details. Finally, we assessed the eligible studies based on the Newcastle Ottawa Scale (NOS). This scale ranges from 0 to 9 and contains nine items (1 point for each) in three parts: selection (four items), comparability (two items) and exposure or outcomes (three items). Scores of 0-3 indicated studies to being of poor quality; scores of 4-6 indicated studies to being of moderate quality; and scores of 7 or higher indicated studies to be of high quality (supplementary box S2).

Statistical analysis

The data extracted from eligible studies were in the form of effect estimates (*OR* or *RR*) and corresponding 95% *CI*s. Due to the low level of morbidity in babies born of

For peer review only - http://bmjopen.bmj.com/site/about/guidelines.xhtml

SGA, the *OR* was approximately equal to the *RR*.[19] Meta-analysis was performed using the STATA package version 12.0 (Stata Corporation, College Station, TX, USA). The *OR*s and 95% *CI*s for normal vitamin D levels versus deficient vitamin D levels from each study were combined to calculate an estimated pooled *OR*, *95% CI*, and *P* value. The *Q*-statistic test and I-squared (I^2) test were used to estimate the heterogeneity among studies.[20] The random effects model is usually more suitable when study data are gathered from the published literature.[21] Therefore, the random effects model was used in our meta-analysis. To evaluate the sources of heterogeneity and the various results obtained for pre-specified subgroups, subgroup analysis was performed based on cut-off values, study quality (NOS scores), adjustment for critical confounders, sample size, measurement of vitamin D, and the gestational week in which blood sampling was performed. A sensitivity analysis was conducted to determine the stability and reliability of the results by omitting one study at a time and confirming the consistency of the overall effect estimate. Funnel plots were used to qualitatively assess the publication bias, whereas Egger's and Begg's tests were used to quantitatively assess publication bias.[22 23]

RESULTS

Description of included studies

A total of 1734 studies were identified for initial review using the described search strategies. After removing duplicates, 1536 studies remained. We screened the titles and abstracts of these studies and excluded 1518 records according to the inclusion and exclusion criteria. The 18 remaining full-text articles were then assessed for eligibility. Finally, 13 cohort studies[4 15-18 24-31] were included in the meta-analysis (figure 1), with a total sample of 28 285 pregnant women.

The characteristics and methodological quality of the 13 studies are presented in table 1 and supplementary table S1. These studies were published between 2010 and 2016; four were conducted in the United states, three in the Netherlands, two in China and one each in Korea, Singapore, Ireland, and New Zealand. The average age of the

For peer review only - http://bmjopen.bmj.com/site/about/guidelines.xhtml

pregnant women in these studies was <30 years for four studies and >30 years for five studies; the average pre-pregnancy BMI of the participants was <25 kg/m^2 in seven studies and >25 kg/m^2 in three studies. Ten studies adjusted for confounders and three studies have not. Five studies collected blood during the first trimester, five during the second trimester, and three during a mixture of the first, second, and third trimesters. Five assay methods were used to measure the vitamin D levels of pregnant women, and two criteria were used for the diagnosis of SGA infants (birthweight in the lowest 10th or 15th percentile of the reference population). The prevalence of maternal vitamin D deficiency varied from 13.2% to 77.3% (supplementary table S1). NOS scores were presented as either representing high levels (nine studies) or low levels (four studies) (supplementary table S2).

Meta-analysis results

The overall results revealed that maternal vitamin D deficiency during pregnancy was significantly associated with an increased risk of SGA infants (pooled *OR* = 1.588; *95% CI* 1.138 to 2.216; $P < 0.01$) in the random effects model. A forest plot showing the details is presented in figure 2.

Subgroup analysis

Due to the existence of heterogeneity ($I^2 = 84.2\%$; $P < 0.001$), subgroup analysis was performed to investigate the possible sources of heterogeneity in the meta-analysis (table 2). The subgroups were created based on cut-off vitamin D levels, measurement of vitamin D, sample size, study quality (NOS score), whether the study adjusted for critical confounders, and the gestational week in which blood sampling was performed. In subgroup analyses, the confidence intervals for each subgroup was overlapped, indicating no significant differences in the effect estimates. Thus, there were no differences in the association between vitamin D deficiency and SGA infants based on study quality, time of blood sampling, cut-off vitamin D levels, sample size, adjustment for critical confounders, and measurement of vitamin D (table 2). However, we did not conduct subgroup analyses regarding ethnicity, pre-pregnancy

For peer review only - http://bmjopen.bmj.com/site/about/guidelines.xhtml

BMI, gestational age of infant at birth, and season during which blood sampling was performed due to insufficient or unspecific data in some studies.

Sensitivity analysis and publication bias

To evaluate the stability of our results, sensitivity analysis was performed. Chen's study[4] was discovered to be responsible for most of the heterogeneity in this meta-analysis. Excluding that study resulted in low heterogeneity among the remaining studies (I^2 = 55.4%, *P* = 0.010) with a pooled *OR* of 1.336 (95% *CI* 1.103 to 1.692). Furthermore, there were no obvious changes in the pooled *ORs* as a result of the exclusion of any other single study; the pooled *OR*s obtained ranged from 1.366 (95% *CI* 1.103 to 1.692) to 1.693 (95% *CI* 1.211 to 2.366), and each was statistically significant (table 3). Additionally, no publication bias was identified using Begg's test (*P* = 0.669) and Egger's regression test (*P* = 0.815). A funnel plot displaying the details is presented in figure 3.

DISCUSSION

The prevalence of vitamin D deficiency during pregnancy and its association with the risk of SGA infants are attracting increasing attentions. The present meta-analysis of prospective cohort studies suggested that vitamin D deficiency is significantly associated with a higher risk of SGA. No publication bias was detected, and sensitivity analysis demonstrated that no single study markedly affected the results, which indicated that the results of our meta-analysis are stable and reliable.

The findings of our study are in agreement with several previous studies. One previous meta-analysis showed that a low maternal vitamin D levels during pregnancy may be associated with an increased risk of SGA, gestational diabetes mellitus, and preterm birth.[5] Similarly, another vital meta-analysis suggested that vitamin D insufficiency was associated with an increased risk of SGA, preeclampsia, and bacterial vaginosis.[6] However, those meta-analyses included both case-control and prospective cohort studies and did not include the most recently published cohort studies; additionally, they did not evaluate the association using specific subgroup

For peer review only - http://bmjopen.bmj.com/site/about/guidelines.xhtml

analysis. Moreover, the cut-off vitamin D levels differed between different studies. Thus, we conducted this meta-analysis to provide stronger evidence for the association between vitamin D and SGA.

The heterogeneity test (Cochran *Q* test) revealed significant heterogeneity among the studies in this meta-analysis. We investigated the potential factors affecting the results by performing subgroup analysis. The results of the subgroup analyses demonstrated no significant differences in the association between vitamin D deficiency and SGA based on study quality, gestational week during which blood sampling was performed, cut-off values, sample size, adjustment for critical confounders and measurement of vitamin D; however, other factors may have contributed to the heterogeneity in our meta-analysis. Maternal ethnicity, season during which blood sampling was performed, and sunlight exposure and diet during pregnancy are confounding factors for the association between vitamin D deficiency and SGA. Sensitivity analysis revealed that exclusion of any single study did not materially alter the overall combined effect, but also that Chen's study[4] probably contributed greatly to the heterogeneity observed. Therefore, we should interpret the results of this meta-analysis objectively.

The underlying mechanism through which vitamin D deficiency increases the risk of SGA infants is not entirely clear but may be related to the inflammatory response. Vitamin D deficiency can increase levels of proinflammatory cytokines, leading to oxidative stress. Lower 25(OH)D status is associated with increased vascular endothelial cell expression of nuclear factor κB (NFκB) and interleukin 6 and with decreased expression of vitamin D receptor and 1-α hydroxylase.[32] One study reported that levels of pro-inflammatory cytokines in the cord blood of SGA infants were significantly higher than those in the cord blood of non-SGA infants.[33] Mullins et al.[34] reported that more tumor necrosis factor (TNF-α) was expressed in pregnant women who born SGA infants than normal infants of pregnant women, and as a critical inflammatory factor, TNF-α was previously revealed to inhibit placental hormone synthesis and stimulate calcitriol catabolism through the regulation of enzymes.[35]

For peer review only

For peer review only - http://bmjopen.bmj.com/site/about/guidelines.xhtml

Vitamin D may also play a crucial role in innate and adaptive immunity by inhibiting the decidual NFκB pathway to reduce inflammatory response, because NFκB is a main transcription factor of inflammatory mediators.[36]

Maternal vitamin D deficiency is common and is influenced by numerous variables including ethnicity, region of residence, skin pigmentation, sun exposure, season, age, and vitamin D supplementation.[37] The American Association of Endocrinology states that pregnant women require at least 600 IU/d of vitamin D and that at least 1500-2000 IU/d of vitamin D may be necessary to maintain a blood level of >30 ng/mL.[38] However, recommendations of vitamin D supplementation for pregnant women are scant. Vitamin D supplementation during pregnancy was suggested as an intervention to prevent adverse pregnancy outcomes.[39] A randomized controlled trial reported that maternal vitamin D supplementation of 2000 or 4000 IU/d appeared to be safe during pregnancy, and the most effective supplementation for optimizing serum vitamin D concentrations in mothers and their infants was 4000 IU/d.[40] This result is consistent with another randomized controlled trial in Pakistan.[41] In two studies, low vitamin D levels during pregnancy increased the risk of SGA, however, vitamin D supplementation did not significantly reduce the risk of SGA (*OR* = 0.78, *95% CI* 0.50 to 1.21[42] and *OR* = 0.67,95% *CI* 0.40 to 1.11[43]). Another study found it difficult to draw a final conclusion regarding the need for vitamin D supplementation during pregnancy.[44] Therefore, larger randomized controlled trials are required to assess the value of such interventions, and will have a significant impact on the guidance regarding perinatal care.

Our study had several strengths. First, to ensure that evidence was reliable, we included only prospective cohort studies, which have more advantages than case control studies. Second, no publication bias was present in our meta-analysis, indicating that its results may be unbiased and credible. Finally, our study's subgroup analysis enabled thorough understanding of the current evidence. However, several limitations should also be acknowledged. The association between maternal vitamin D status and SGA risk may have been affected by confounding factors such as

For peer review only

For peer review only - http://bmjopen.bmj.com/site/about/guidelines.xhtml

pre-pregnancy BMI, age, education, ethnicity, and sunlight exposure; not all the included studies controlled for these confounding factors. Additionally, the included studies had different definitions of vitamin D deficiency, insufficiency, or sufficiency, which may have affected the results. Lastly, pooled data without detailed individual information were used to perform the meta-analysis, which restricted us from obtaining comprehensive results.

CONCLUSIONS

The present study indicates that a low vitamin D levels is associated with an increased risk of SGA infants. Further confirmation of these findings in larger-sample size studies is required. The role of vitamin D in the pathogenesis of SGA should be emphasized. Additionally, early screening for vitamin D deficiency among pregnant women may be necessary.

Contributors FT contributed to study design; SL, XW and BZ contributed analysis tools and methods; YC analyzed the data and drafted the manuscript; BZ and FT revised the manuscript. All authors read and approved the final version of the manuscript.

Funding This work is grateful for the financial support offered by the National Natural Science Foundation of China (81330068, 81573168)

Data sharing statement No additional data are available

Competing interests None declared.

For peer review only

REFERENCES

1. Finberg L. Vitamin D deficiency and rickets. *J Pediatr Endocrinol Metab* 2006; 19:203.
2. Palacios C, Gonzalez L. Is vitamin D deficiency a major global public health problem? *J Steroid Biochem Mol Biol* 2014; 144 Pt A:138-145.
3. Eggemoen AR, Falk RS, Knutsen KV, *et al*. Vitamin D deficiency and supplementation in pregnancy in a multiethnic population-based cohort. *BMC pregnancy and childbirth* 2016; 16:7.
4. Chen YH, Fu L, Hao JH, *et al*. Maternal vitamin D deficiency during pregnancy elevates the risks of small for gestational age and low birth weight infants in Chinese population. *J Clin Endocrinol Metab* 2015; 100:1912-1919.
5. Wei SQ, Qi HP, Luo ZC, *et al*. Maternal vitamin D status and adverse pregnancy outcomes: a systematic review and meta-analysis. *J Matern Fetal Neonatal Med* 2013; 26:889-899.
6. Aghajafari F, Nagulesapillai T, Ronksley PE, *et al*. Association between maternal serum 25-hydroxyvitamin D level and pregnancy and neonatal outcomes: systematic review and meta-analysis of observational studies. *BMJ* 2013; 346:f1169.
7. Mikolajczyk RT, Zhang J, Betran AP, *et al*. A global reference for fetal-weight and birthweight percentiles. *Lancet* 2011; 377:1855-1861.
8. Hoftiezer L, Hukkelhoven CW, Hogeveen M, *et al*. Defining small-for-gestational-age: prescriptive versus descriptive birthweight standards. *Eur J Pediatr* 2016; 175:1047-1057.
9. Ding G, Tian Y, Zhang Y, *et al*. Application of a global reference for fetal-weight and birthweight percentiles in predicting infant mortality. *BJOG* 2013; 120(13):1613-1621.
10. Christian P, Murray-Kolb LE, Tielsch JM, *et al*. Associations between preterm birth, small-for-gestational age, and neonatal morbidity and cognitive function among school-age children in Nepal. *BMC pediatrics* 2014; 14:58.
11. Katz J, Lee AC, Kozuki N, *et al*. Mortality risk in preterm and small-for-gestational-age infants in low-income and middle-income countries: a pooled country analysis. *Lancet* 2013; 382(9890):417-425.
12. de Zegher F, Sebastiani G, Diaz M, *et al*. Body composition and circulating high-molecular-weight adiponectin and IGF-I in infants born small for gestational age: breast- versus formula-feeding. *Diabetes* 2012; 61:1969-1973.
13. Wolfenstetter A, Simonetti GD, Poschl J, *et al*. Altered cardiovascular rhythmicity in children born small for gestational age. *Hypertension* 2012; 60:865-870.
14. Aly H, Davies J, El-Dib M, *et al*. Renal function is impaired in small for gestational age premature infants. *J Matern Fetal Neonatal Med* 2013; 26:388-391.
15. Leffelaar ER, Vrijkotte TG, van Eijsden M. Maternal early pregnancy vitamin D status in relation to fetal and neonatal growth: results of the multi-ethnic Amsterdam Born Children and their Development cohort. *Br J Nutr* 2010; 104:108-117.
16. Gernand AD, Simhan HN, Klebanoff MA, *et al*. Maternal serum 25-hydroxyvitamin D and measures of newborn and placental weight in a U.S. multicenter cohort study. *J Clin Endocrinol Metab* 2013; 98:398-404.
17. Nobles CJ, Markenson G, Chasan-Taber L. Early pregnancy vitamin D status and risk for adverse maternal and infant outcomes in a bi-ethnic cohort: the Behaviors Affecting Baby and You (B.A.B.Y.) Study. *Br J Nutr* 2015; 114:2116-2128.

For peer review only

For peer review only - http://bmjopen.bmj.com/site/about/guidelines.xhtml

18. Ong YL, Quah PL, Tint MT, *et al*. The association of maternal vitamin D status with infant birth outcomes, postnatal growth and adiposity in the first 2 years of life in a multi-ethnic Asian population: the Growing Up in Singapore Towards healthy Outcomes (GUSTO) cohort study. *Br J Nutr* 2016, 116:621-631.

19. Gagne JJ, Power MC. Anti-inflammatory drugs and risk of Parkinson disease: a meta-analysis. *Neurology* 2010; 74:995-1002.

20. Takkouche B, Cadarso-Suarez C, Spiegelman D. Evaluation of old and new tests of heterogeneity in epidemiologic meta-analysis. *Am J Epidemiol* 1999; 150:206-215.

21. Borenstein M, Hedges LV, Higgins JP, *et al*. A basic introduction to fixed-effect and random-effects models for meta-analysis. *Res Synth Methods* 2010; 1(2):97-111.22. Egger M, Davey Smith G, Schneider M, *et al*. Bias in meta-analysis detected by a simple, graphical test. *BMJ* 1997; 315:629-634.

23. Begg CB, Mazumdar M. Operating characteristics of a rank correlation test for publication bias. *Biometrics* 1994; 50:1088-1101.

24. Burris HH, Rifas-Shiman SL, Camargo CA, *et al*. Plasma 25-hydroxyvitamin D during pregnancy and small-for-gestational age in black and white infants. *Ann Epidemiol* 2012; 22:581-586.

25. Zhou J, Su L, Liu M, *et al*. Associations between 25-hydroxyvitamin D levels and pregnancy outcomes: a prospective observational study in southern China. *Eur J Clin Nutr* 2014; 68:925-930.

26. Choi R, Kim S, Yoo H, *et al*. High prevalence of vitamin D deficiency in pregnant Korean women: the first trimester and the winter season as risk factors for vitamin D deficiency. *Nutrients* 2015; 7:3427-3448.

27. Kiely ME, Zhang JY, Kinsella M, *et al*. Vitamin D status is associated with uteroplacental dysfunction indicated by pre-eclampsia and small-for-gestational-age birth in a large prospective pregnancy cohort in Ireland with low vitamin D status. *Am J Clin Nutr* 2016; 104:354-361.

28. Scholl TO, Chen X, Stein TP. Maternal calcium metabolic stress and fetal growth. *Am J Clin Nutr* 2014; 99:918-925.

29. Boyle VT, Thorstensen EB, Mourath D, *et al*. The relationship between 25-hydroxyvitamin D concentration in early pregnancy and pregnancy outcomes in a large, prospective cohort. *Br J Nutr* 2016; 116:1409-1415.

30. van den Berg G, van Eijsden M, Vrijkotte TG, *et al*. Suboptimal maternal vitamin D status and low education level as determinants of small-for-gestational-age birth weight. *Eur J Nutr* 2013; 52:273-279.

31. Miliku K, Vinkhuyzen A, Blanken LM, *et al*. Maternal vitamin D concentrations during pregnancy, fetal growth patterns, and risks of adverse birth outcomes. *Am J Clin Nutr* 2016; 103:1514-1522.

32. Jablonski KL, Chonchol M, Pierce GL, *et al*. 25-Hydroxyvitamin D deficiency is associated with inflammation-linked vascular endothelial dysfunction in middle-aged and older adults. *Hypertension* 2011; 57:63-69.

33. Lausten-Thomsen U, Olsen M, Greisen G, *et al*. Inflammatory markers in umbilical cord blood from small-for-gestational-age newborns. *Fetal Pediatr Pathol* 2014; 33:114-118.

34. Mullins E, Prior T, Roberts I, *et al*. Changes in the maternal cytokine profile in pregnancies complicated by fetal growth restriction. *Am J Reprod Immunol* 2012; 68:1-7.

For peer review only

35. Diaz L, Noyola-Martinez N, Barrera D, *et al*. Calcitriol inhibits TNF-alpha-induced inflammatory cytokines in human trophoblasts. *J Reprod Immunol* 2009; 81:17-24.

36. Cohen-Lahav M, Shany S, Tobvin D, *et al*. Vitamin D decreases NFkappaB activity by increasing IkappaBalpha levels. *Nephrol Dial Transplant* 2006; 21:889-897.

37. Bodnar LM, Simhan HN. Vitamin D may be a link to black-white disparities in adverse birth outcomes. *Obstet Gynecol Surv* 2010; 65:273-284.

38. Holick MF, Binkley NC, Bischoff-Ferrari HA, *et al*. Evaluation, treatment, and prevention of vitamin D deficiency: an Endocrine Society clinical practice guideline. *J Clin Endocrinol Metab* 2011; 96:1911-1930.

39. De-Regil LM, Palacios C, Lombardo LK, *et al*. Vitamin D supplementation for women during pregnancy. *Sao Paulo Med* 2016; 134:274-275.

40. Dawodu A, Saadi HF, Bekdache G, *et al*. Randomized controlled trial (RCT) of vitamin D supplementation in pregnancy in a population with endemic vitamin D deficiency. *J Clin Endocrinol Metab* 2013; 98:2337-2346.

41. Hossain N, Kanani FH, Ramzan S, *et al*. Obstetric and neonatal outcomes of maternal vitamin D supplementation: results of an open-label, randomized controlled trial of antenatal vitamin D supplementation in Pakistani women. *J Clin Endocrinol Metab* 2014; 99:2448-2455.

42. Perez-Lopez FR, Pasupuleti V, Mezones-Holguin E, *et al*. Effect of vitamin D supplementation during pregnancy on maternal and neonatal outcomes: a systematic review and meta-analysis of randomized controlled trials. *Fertil Steril* 2015; 103:1278-1288.

43. Thorne-Lyman A, Fawzi WW. Vitamin D during pregnancy and maternal, neonatal and infant health outcomes: a systematic review and meta-analysis. *Paediatr Perinat Epidemiol* 2012; 26:75-90

44. Karras SN, Fakhoury H, Muscogiuri G, *et al*. Maternal vitamin D levels during pregnancy and neonatal health: evidence to date and clinical implications. *Ther Adv Musculoskelet Dis* 2016; 8(4):124-135.

For peer review only

For peer review only - http://bmjopen.bmj.com/site/about/guidelines.xhtml

Figure legends:

Figure 1. Flowchart of the literature search and trial selection process.

Figure 2. Forest plots of summary crude odds ratios of the association between vitamin D deficiency.

Figure 3. Funnel plot for small for gestational age. Log OR of the individual studies plotted against the standard error of log OR.

For peer review only - http://bmjopen.bmj.com/site/about/guidelines.xhtml

Table 1. Characteristics of the included studies in the present meta-analysis

Author	Region	Year	Age at baseline (mean, year)	Pre-pregnancy BMI (mean, kg/m^2)	Gestational week of blood sampling	Measurement of vitamin D	SGA criteria	Cut-off values	Ethnicity group	*OR* (*95% CI*)	Adjusted	NOS Score	Sample size
Leffelaar[15]	Netherlands	2010	NA	NA	12-14 weeks	enzyme immunoassay	<10th	< 15 ng/ml	Dutch (60.3%), Surinamese (6.7%), Turkish (4.0%), Moroccan (6.3%), Other non-western (14.2%), Other western (8.6%)	1.90 (1.40,2.70)	yes	8	3730
Burris[24]	USA	2012	32.5	24.8	26-28 weeks	CLIA and RIA	<10th	< 10 ng/ml	White (83.6%), Black (16.4%)	3.17 (1.16,8.63)	yes	7	1133
Zhou[25]	China	2014	29.5	20.3	16-20 weeks	ECLIA	<10th	< 20 ng/ml	Asian	2.46 (0.71,8.46)	no	8	1923
Choi[26]	Korea	2015	32.0	20.2	first or second or third trimester	LC-MS/MS	<10th	< 20 ng/ml	Asian	0.448 (0.149,1.351)	yes	6	220
Ong[18]	Singapore	2016	30.5	26.1	26-28 weeks	LC-MS/MS	<10th	< 20 ng/ml	Asian	1.00 (0.56,1.79)	yes	8	910
Kiely[27]	Ireland	2016	30.5	24.9	14-16 weeks	LC-MS/MS	<10th	< 20 ng/ml	White (98%), Others (2%)	0.88 (0.60,1.28)	yes	6	1768
Scholl[28]	USA	2014	22.8	26	13.8±5.6 weeks	HPLC	<10th	< 20 ng/ml	Hispanic (51.4%), Non-Hispanic black (34.4%), Non-Hispanic white (14.2%)	0.930 (0.568,1.523)	no	8	1045
Chen[4]	China	2015	27.5	NA	first or second or third trimester	RIA	<10th	< 20 ng/ml	Asian	6.47 (4.30,9.75)	yes	6	3658
Boyle[29]	New Zealand	2016	30.3	24.8	15 weeks	LC-MS/MS	<10th	< 20 ng/ml	NZ European (83.8%), other ethnicities (16.2%)	1.33 (0.91,1.96)	yes	7	2065
Berg[30]	Netherlands	2013	NA	NA	12.9 weeks	enzyme immunoassay	<10th	< 20 ng/ml	NA	1.57 (1.03,2.39)	yes	7	2274
Gerand[16]	USA	2013	NA	22.3	20.6 weeks	LC-MS/MS	<10th	<15 ng/ml	White (52.1%), Black (41.6%), Puerto Rican (6.3%)	1.284 (1.026,1.608)	no	6	2146
Miliku[31]	Netherlands	2016	29.7	23.7	20.3 weeks	LC-MS/MS	<15th	<10 ng/ml	European (57.3%), Cape Verdean (4.4%), Dutch Antillean (3.5%), Moroccan (6.6%), Surinamese (9.1%), Turkish (9.2%), Other (9.9%)	2.07 (1.33,3.22)	yes	7	7176
Nobles[17]	USA	2015	NA	>25	first or second or third trimester	ECLIA	<10th	< 20 ng/ml	[White (75.6%), Black (13.5%)]	2.14 (0.67,6.88)	yes	8	237

For peer review only - http://bmjopen.bmj.com/site/about/guidelines.xhtml

CLIA: chemiluminescence immunoassay; RIA: radioimmunoassay; ECLIA: electrochemiluminescence immunoassay; LC-MS/MS: liquid chromatography-tandem mass spectrometry; NA: not available.

Table 2. Subgroup analysis of the association between maternal Vitamin D deficiency and SGA

Stratification group	N	*P* Value for *OR*	*OR (95% CI)*	Heterogeneity test	
				I-square (%)	*P* Value
Study quality (NOS)					
High	9[15 17 18 24 25 28-31]	<0.001	1.555 (1.239, 1.951)	37.6	0.118
Low	4[4 16 26 27]	0.440	1.441 (0.570, 3.641)	95.2	<0.001
Gestation of blood sampling					
first trimester	5[15 27 28 29 30]	0.104	1.286 (0.950,1.741)	65.9	0.020
second trimester	5[16 18 24 25 31]	0.011	1.577 (1.110, 2.240)	51.1	0.085
Mixed (first or second or third)	3[4 17 26]	0.432		90.6	<0.001
Cut-off values					
＜10 ng/ml	2[24 31]	0.001	2.219 (1.480, 3.325)	0	0.446
＜15 ng/ml	2[15 16]	0.029	1.532 (1.046, 2.246)	73.2	0.054
＜20 ng/ml	9[4 17 18 25-30]	0.172	1.448 (0.851, 2.465)	88.2	＜0.001
Sample size					
> 1000	10[4 15 16 24 25 27-31]	0.003	1.760 (1.217, 2.544)	86.8	<0.001
< 1000	3[17 18 26]	0.946	0.975 (0.476, 1.999)	45.5	0.160
Adjust for critical confounders					
yes	10[4 15 17 18 24 26 29-31]	0.018	1.681 (1.094, 2.584)	86.3	< 0.001
no	3[16 25 28]	0.180	1.219 (0.912, 1.629)	22.3	0.276

For peer review only

Measurement of vitamin D					
LC-MS/MS	6[16 18 26 27 29 31]	0.204	1.195 (0.908, 1.573)	59.5	0.031
Others	7[4 15 17 24 25 28 29]	0.006	2.224 (1.263, 3.918)	85.8	<0.001

Table 3. Sensitivity analyses of the association between vitamin D deficiency and SGA

**Study omitted**	***OR* (95% CI)**	***P* value**	**I-square (%)**	***P* value**
Leffelaar[15]	1.559 (1.074, 2.263)	0.020	85.2	< 0.001
Burris[24]	1.527 (1.084, 2.152)	0.016	85.1	< 0.001
Zhou[25]	1.557 (1.105, 2.195)	0.011	85.4	< 0.001
Choi[26]	1.693 (1.211, 2.366)	0.002	84.5	< 0.001
Ong[18]	1.652 (1.162, 2.350)	0.005	85.0	< 0.001
Kiely[27]	1.686 (1.191, 2.387)	0.003	83.4	< 0.001
Scholl[28]	1.669 (1.174, 2.371)	0.004	84.6	< 0.001
Chen[4]	1.366 (1.103, 1.692)	0.004	55.4	0.010
Boyle[29]	1.616 (1.118, 2.335)	0.011	85.4	< 0.001
Berg[30]	1.590 (1.102, 2.293)	0.013	85.4	< 0.001
Gerand[16]	1.624 (1.100, 2.397)	0.015	84.7	< 0.001

For peer review only

For peer review only - http://bmjopen.bmj.com/site/about/guidelines.xhtml

Miliku[31]	1.548 (1.079, 2.220)	0.018	85.1	< 0.001
Nobles[17]	1.565 (1.109, 2.209)	0.011	85.4	< 0.001

For peer review only

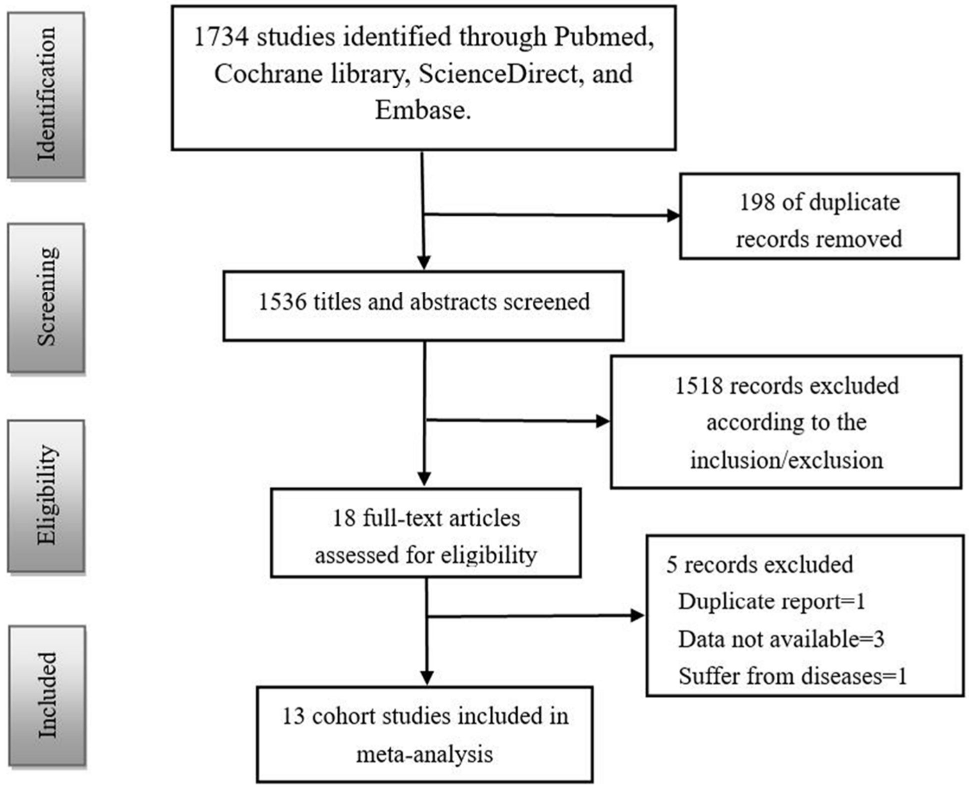

Figure 1. Flowchart of the literature search and trial selection process

129x105mm (300 x 300 DPI)

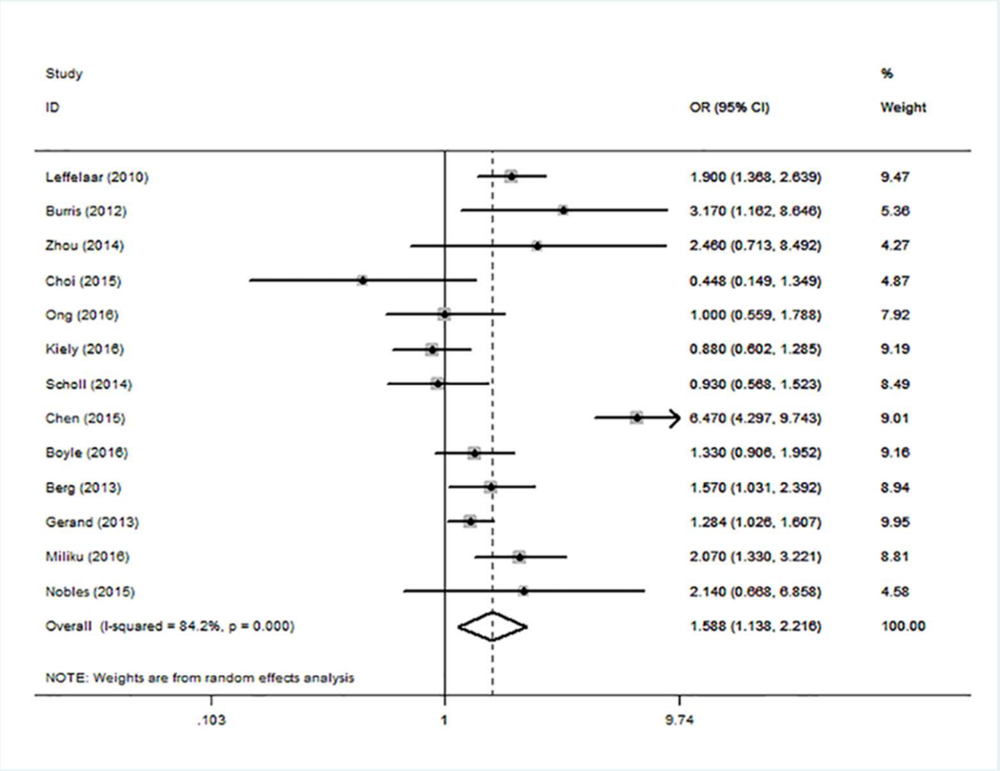

Figure 2. Forest plots of summary crude odds ratios of the association between vitamin D deficiency

91x70mm (300 x 300 DPI)

For peer review only

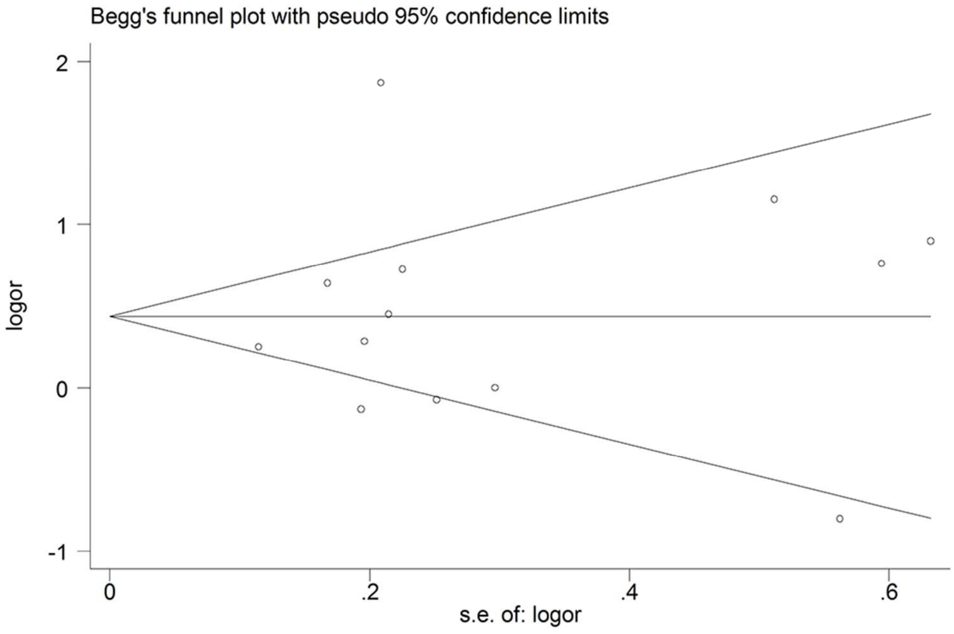

Figure 3. Funnel plot for small for gestational age. Log OR of the individual studies plotted against the standard error of log OR.

91x60mm (300 x 300 DPI)

view only

Supplementary Box S1. The search strategy of PubMed

Source: PubMed

Search on: February 28th, 2017

#1 ((("vitamin D"[Mesh] OR "cholecalciferol"[Mesh]) OR "25-hydroxyvitamin D"[Title/Abstract]) OR "25(OH)D"[Title/Abstract])

#2 ((("small for gestational age"[Title/Abstract] OR "small-for-gestation-age"[Title/Abstract]) OR "small size for gestational age"[Title/Abstract]) OR SGA [Title/Abstract])

#3 #1 AND #2

The search strategy in other databases did some adjustments on the basis of the above database.

For peer review only

Supplementary Table S1. Characteristics of the included studies in the present meta-analysis

Author	Latitude	The time of year data collected	Gestational age of infant at birth	The prevalence of SGA	The prevalence of vitamin D deficiency	Maternal education status	Season of blood sample
Leffelaar[15]	NA	2003.2~2004.3	40.1±1.2 weeks	9.2%	23.1%	≤5 years (17.2%), 6-10years (38.5%), ≥11 years (44.3%)	Summer (43.6%)
Burris[24]	NA	NA	39.6 weeks	4.8%	32.4%	College graduate (41.2%)	NA
Zhou[25]	23.1°N	2010.9~2011.8	NA	0.6%	18.9%	NA	NA
Choi[26]	36.0°N	2012.4~2013.9	NA	10.9%	77.3%	≤12 years(5.5%), > 12 years(94.5%)	Spring (44.5%), Summer (10.0%), Fall (39.5%), Winter (5.9%)
Ong[18]	1°22′N	NA	NA	9.1%	13.2%	Primary and secondary (30.2%), Post-secondary (35.4%), University (34.4%)	NA
Kiely[27]	52°N	2008.3~2011.2	NA	10.7%	44.0%	Secondary (61%), Tertiary (39%)	Winter (58.5%), Summer (41.5%)
Scholl[28]	NA	2001~2007	38.5	7.2%	33.7%	NA	NA
Chen[4]	31°52′N	2008.11~2010.10	NA	8.9%	38.41%	NA	Spring (36.7%), Summer (22.5%), Fall (20.6%), Winter (20.2%)
Boyle[29]	NA	2005~2008	NA	9.9%	21.5%	NA	Spring (20.5%), Summer (26.4%), Fall (23.2%), Winter (29.8%)
Berg[30]	52°22′N	2003.2~2004.3	20-42 weeks	9.1%	NA	NA	Winter (55.5%)
Gerand[16]	≥41°N(63.0%), 38~40°N(28.8%), ≤35°N(8.2%)	1959~1965	39.7±1.3 weeks	18.4%	34.8%	NA	Spring (25.9%), Summer (25.7%), Fall (24.6%), Winter (23.9%)
Miliku[31]	NA	2002.4~2006.1	35.9-42.3 weeks	5.0%	53.2%	No higher education (59.2%), Higher education (40.8%)	Spring (29.5%), Summer (22.9%), Fall (24.0%), Winter (23.6%)
Nobles[17]	NA	2007~2012	NA	9.6%	20.7%	≤High school (55.2%), ＞High school (44.8%)	Summer (41.4%), Winter (58.7%)

NA：not available.

For peer review only

Supplementary Box S2. Quality assessment of cohort studies

NEWCASTLE - OTTAWA QUALITY ASSESSMENT SCALE

COHORT STUDIES

Note: A study can be awarded a maximum of one star for each numbered item within the Selection and Outcome categories. A maximum of two stars can be given for Comparability

Selection

1) Representativeness of the exposed cohort

a) truly representative of the average _______________ (describe) in the community ✵

b) somewhat representative of the average ______________ in the community ✵

c) selected group of users eg nurses, volunteers

d) no description of the derivation of the cohort

2) Selection of the non-exposed cohort

a) drawn from the same community as the exposed cohort ✵

b) drawn from a different source

c) no description of the derivation of the non-exposed cohort

3) Ascertainment of exposure

a) secure record (eg surgical records) ✵

b) structured interview ✵

c) written self-report

d) no description

4) Demonstration that outcome of interest was not present at start of study

a) yes ✵

b) no

Comparability

1) Comparability of cohorts on the basis of the design or analysis

a) study controls for _____________ (select the most important factor) ✵

b) study controls for any additional factor ✵ (This criteria could be modified to indicate specific control for a second important factor.)

Outcome

1) Assessment of outcome

a) independent blind assessment ✵

b) record linkage ✵

c) self-report

d) no description

2) Was follow-up long enough for outcomes to occur

a) yes (select an adequate follow up period for outcome of interest) ✵

b) no

3) Adequacy of follow up of cohorts

a) complete follow up - all subjects accounted for ✵

b) subjects lost to follow up unlikely to introduce bias - small number lost - > ____ % (select an adequate %) follow up, or description provided of those lost) ✵

c) follow up rate < ____% (select an adequate %) and no description of those lost

d) no statement

For peer review only

Supplementary Table S2. Quality scores of included studies on vitamin D status and SGA.

Study	Selection				Comparability	Outcome			Total scores
	Representativeness of the exposed cohort	Selection of the non-exposed cohort	Ascertainment of exposure	Demonstration that outcome of interest was not present at start of study	Comparability of cohorts on the basis of design or analysis	Assessment of outcome	Was follow-up long enough for outcomes to occur	Adequacy of follow up of cohorts	
Leffelaar[14]	1	1	1	1	1	1	1	1	8
Burris[23]	1	1	1	1	1	1	1	0	7
Zhou[24]	1	1	1	1	1	1	1	1	8
Choi[25]	1	1	1	1	1	1	0	0	6
Ong[17]	1	1	1	1	2	1	1	0	8
Kiely[26]	1	0	1	1	1	1	1	0	6
Scholl[27]	1	1	1	1	2	0	1	1	8
Chen[4]	1	1	1	1	0	1	1	0	6
Boyle[28]	1	1	1	1	1	1	1	0	7
Berg[29]	1	1	1	1	1	1	1	0	7
Gerand[15]	1	1	1	1	0	1	1	0	6
Miliku[30]	1	1	1	1	1	1	1	0	7
Nobles[16]	1	1	1	1	1	1	1	1	8

For peer review only

PRISMA 2009 Checklist

Section/topic	#	Checklist item	Reported on page #
**TITLE**			
Title	1	Identify the report as a systematic review, meta-analysis, or both.	P1: Line 1
**ABSTRACT**			
Structured summary	2	Provide a structured summary including, as applicable: background; objectives; data sources; study eligibility criteria, participants, and interventions; study appraisal and synthesis methods; results; limitations; conclusions and implications of key findings; systematic review registration number.	P2: Line 15-42
**INTRODUCTION**			
Rationale	3	Describe the rationale for the review in the context of what is already known.	P3-4: Line 45-75
Objectives	4	Provide an explicit statement of questions being addressed with reference to participants, interventions, comparisons, outcomes, and study design (PICOS).	P3-4: Line 45-75
**METHODS**			
Protocol and registration	5	Indicate if a review protocol exists, if and where it can be accessed (e.g., Web address), and, if available, provide registration information including registration number.	No registration
Eligibility criteria	6	Specify study characteristics (e.g., PICOS, length of follow-up) and report characteristics (e.g., years considered, language, publication status) used as criteria for eligibility, giving rationale.	P4-P5: Line 89-104
Information sources	7	Describe all information sources (e.g., databases with dates of coverage, contact with study authors to identify additional studies) in the search and date last searched.	P4: Line 78-84
Search	8	Present full electronic search strategy for at least one database, including any limits used, such that it could be repeated.	P4: Line 82-84 (supplementary Box S1)
Study selection	9	State the process for selecting studies (i.e., screening, eligibility, included in systematic review, and, if applicable, included in the meta-analysis).	figure 1
Data collection process	10	Describe method of data extraction from reports (e.g., piloted forms, independently, in duplicate) and any processes for obtaining and confirming data from investigators.	P5: Line 105-125
Data items	11	List and define all variables for which data were sought (e.g., PICOS, funding sources) and any assumptions and simplifications made.	P5: Line 109-125
Risk of bias in individual studies	12	Describe methods used for assessing risk of bias of individual studies (including specification of whether this was done at the study or outcome level), and how this information is to be used in any data synthesis.	P5: Line 119-125 (supplementary Box S2)
Summary measures	13	State the principal summary measures (e.g., risk ratio, difference in means).	P5-6: Line 114-133
Synthesis of results	14	Describe the methods of handling data and combining results of studies, if done, including measures of consistency (e.g., I^2) for each meta-analysis.	P5-6: Line 127-144

For peer review only

For peer review only - http://bmjopen.bmj.com/site/about/guidelines.xhtml

PRISMA 2009 Checklist

Section/topic	#	Checklist item	Reported on page #
Risk of bias across studies	15	Specify any assessment of risk of bias that may affect the cumulative evidence (e.g., publication bias, selective reporting within studies).	P6: Line 140-144
Additional analyses	16	Describe methods of additional analyses (e.g., sensitivity or subgroup analyses, meta-regression), if done, indicating which were pre-specified.	P6: Line 133-144
**RESULTS**			
Study selection	17	Give numbers of studies screened, assessed for eligibility, and included in the review, with reasons for exclusions at each stage, ideally with a flow diagram.	P6: Line 147-152 (figure 1)
Study characteristics	18	For each study, present characteristics for which data were extracted (e.g., study size, PICOS, follow-up period) and provide the citations.	P6-7: Line 153-167 (table 1)
Risk of bias within studies	19	Present data on risk of bias of each study and, if available, any outcome level assessment (see item 12).	Supplementary table S2 and table 3
Results of individual studies	20	For all outcomes considered (benefits or harms), present, for each study: (a) simple summary data for each intervention group (b) effect estimates and confidence intervals, ideally with a forest plot.	table 1 and figure 2
Synthesis of results	21	Present results of each meta-analysis done, including confidence intervals and measures of consistency.	P7: Line 169-172 (figure 2)
Risk of bias across studies	22	Present results of any assessment of risk of bias across studies (see Item 15).	P8: Line 195-197 (figure 3)
Additional analysis	23	Give results of additional analyses, if done (e.g., sensitivity or subgroup analyses, meta-regression [see Item 16]).	P7-8: Line 173-197 (table 2 and table 3)
**DISCUSSION**			
Summary of evidence	24	Summarize the main findings including the strength of evidence for each main outcome; consider their relevance to key groups (e.g., healthcare providers, users, and policy makers).	P8-10: Line 199-263
Limitations	25	Discuss limitations at study and outcome level (e.g., risk of bias), and at review-level (e.g., incomplete retrieval of identified research, reporting bias).	P10-11: Line 268-276
Conclusions	26	Provide a general interpretation of the results in the context of other evidence, and implications for future research.	P11: Line 277-282
**FUNDING**			
Funding	27	Describe sources of funding for the systematic review and other support (e.g., supply of data); role of funders for the systematic review.	P11: Line 287-288

For peer review only
